# Supplementary material for: Selective flexible packaging pathways of the segmented genome of influenza A virus
Source: Nat Commun. 2020 Aug 28;11:4355. doi: 10.1038/s41467-020-18108-1 (PMC7455735; doi:10.1038/s41467-020-18108-1)
Supplement: Supplementary file 1 — Supplementary Information [file 41467_2020_18108_MOESM1_ESM.pdf]

Selective flexible packaging pathways of the segmented genome of Influenza A Virus

Haralampiev et al.

Supplementary Information

Supplementary Method

Calculation of all possible combinations of vRNPs

Considering all possible combinations of vRNPs, the random packaging model predicts that 98% of the MSCs would contain two or more copies of a distinct segment species. This value follows from the equation:

(1- (Combinations without repetitions/Combinations with repetitions)) * 100% (1)

with

Combinations with repetition (up to k copies of a segment can occur in an MSC of rank *k*; *n* – number of different segments (*n* = 8))

$$\frac{(n+k-1)!}{(n-1)!k!} = \binom{n+k-1}{k} = \binom{n+k-1}{n-1} = \left(\!\!\binom{n}{k}\!\!\right) \qquad (2)$$

and

Combinations without repetition (only one copy per segment occur in an MSC of rank *k*; *n* – number of different segments (*n* = 8))

$$\frac{n!}{(n-k)!k!} = \frac{n\,(n-1)\,(n-2)\ldots(n-k+1)}{k!} = \binom{n}{n-k} = \binom{n}{k} \qquad (3)$$

Supplementary Note 1

Imaging of vmRNA

To verify specificity of vRNA probes, we visualized all major vmRNAs (Fig. 1, vmRNA) except for M2 and NS2 (see Mat. and Meth.). Cytosolic vmRNA spots did not significantly colocalize with each other or with vRNAs. It is important to note that probes targeting vmRNA also recognize viral cRNA due to the high similarity of their sequences. vmRNA copy numbers have been reported to be about 100-fold higher than cRNA transcripts, which are almost exclusively localized to the nucleus[1, 2], so the contribution of the latter RNA species to fluorescent spots was ignored. Our analysis of the intracellular localization of vmRNA species revealed differences in their distribution between the nucleus and cytosol (see Fig. 1). vmRNAs of PB2, PB1, PA and NA appeared to predominantly localize to the nucleus (Supplementary Figure 6), while the remaining vmRNAs were either rather equally distributed between the nucleus and cytoplasm (HA, NP, M1) or were largely found in the cytoplasm (NS1). While here we visualized vmRNAs for the sole purpose of demonstrating the specificity of our FISH probes, we observed a different distribution of vmRNAs between the

nucleus and cytoplasm than found in a previous study on A/PR/8/34-infected 293T cells[3]. Namely, the vmRNAs of PB2, HA, NP, M1 and NS1 were found predominantly localized in the cytosol. Further studies are needed to clarify the extent to which vmRNA distributions are dependent on the strain of IAV and/or target cell line used in a study.

Supplementary Note 2

Verification of vRNA spot binning

For an assessment of the colocalization quality, we calculated the center of mass for each MSC according to (here shown for x coordinate):

$$(\text{Center of Mass})_x = 1/N \sum_k x_k \qquad (4)$$

The index k runs over all segments in the MSC. N is the number of segments in the MSC. The center of mass for y coordinates were calculated accordingly. Next, the distance of each spot towards the center could be calculated and plotted in a histogram (Supplementary Figure 12). Most segments showed a distribution with a marked peak relatively close to the center of mass (25-60 nm distance), with a shoulder towards higher distances up to 250 nm. All in all, even though one or more segments frequently showed signs of sub-optimal registration, distances were still within the cylinder volume that allowed colocalization to be detected.

Supplementary Discussion

Potential role of specific vRNPs in MSC assembly

Although we observed this situation only for a few cells, very likely due to the high MOI used in our experiment, of particular interest for understanding the potential role of segments in MSC assembly are those cells in which one vRNP species is of low abundance in comparison to the other vRNPs. In principal, two situations are interesting. First, if any segment is ‘less important’ in the packaging of MSCs, and only becomes relevant in a late step of packaging, such as in the formation of rank 8 MSCs, we would anticipate a u-shaped MSC frequency distribution with a high occupancy of rank 7 complexes and a rather low occupancy of rank 8, due to the short supply of that specific segment. We observed such a u-shaped frequency distribution of MSC ranks for cells with a low fraction of segment 8 (Fig. 3d and h, ‘Cell 29’). This suggests that at least for A/Panama, this segment is dispensable in the segment packaging pathway

and only relevant for the final step of formation of a complete set of segments, that is MSCs of rank 8.

A second interesting situation concerns the case where a segment which plays an important role already in early steps of MSC assembly is of low abundance. In that case we should expect a left-hand distribution with a frequency maximum for low rank MSCs and a decreasing frequency towards high rank MSCs. We observed such cells even more rarely. In addition, these cells exhibited the low abundance of at least two segments, which does not permit a distinction regarding whether both or only one of the segments are important for early steps of MSC assembly. An exception is 'Cell 23' (Fig. 3c,g,k), which exhibited low quantities of both S1 and S8 (Fig. 3g). As S8 seems to play a decisive role only in the formation of MSCs (see above), the left-hand side MSC frequency distribution found for 'Cell 23' (Fig. 3c) points towards an important role of the long vRNP segment S1 in the early and/or intermediate phase of MSC formation. Of course, this conclusion must be taken with great caution as this is the only cell we could evaluate. Nevertheless, these examples illustrate that frequency distribution analysis is a very useful tool to uncover the role of vRNPs in MSC assembly.

Supplementary References

1. Kawakami E*, et al.* Strand-specific real-time RT-PCR for distinguishing influenza vRNA, cRNA, and mRNA. *J Virol Methods* **173**, 1-6 (2011).
2. Shapiro GI, Gurney T, Jr., Krug RM. Influenza virus gene expression: control mechanisms at early and late times of infection and nuclear-cytoplasmic transport of virus-specific RNAs. *J Virol* **61**, 764-773 (1987).
3. Read EK, Digard P. Individual influenza A virus mRNAs show differential dependence on cellular NXF1/TAP for their nuclear export. *J Gen Virol* **91**, 1290-1301 (2010).

Supplementary Figures

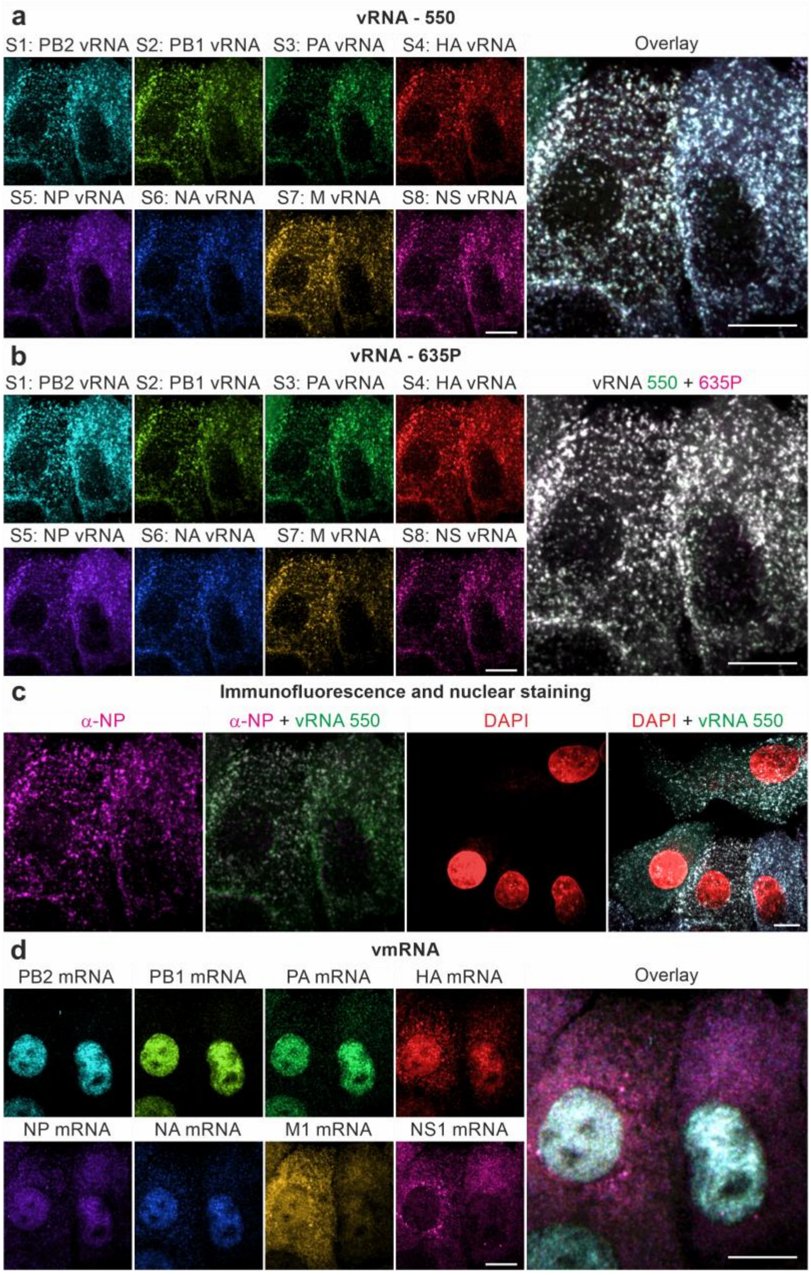

Supplementary Figure 1: Localization of vRNA and vmRNA in A/Panama-infected A549 cells identified by MuSeq-FISH. (a, b) Viral genomic RNAs were stained by FISH 10 h p.i. (MOI 5). To cover all vRNAs and major vmRNAs 12 cycles of FISH labelling were performed. Along these cycles each vRNA was targeted twice, once with with Atto550 (a) (see also Fig. 1) and once with STAR635P (635P) (b) labelled probe sets (for details see text). (c) Immunofluorescence staining of NP. DAPI labelling was performed to exclude nuclear vRNA spots for further colocalization analysis. (d) All major unspliced vmRNAs were stained. Images represent max-z-projections. The intensities of images were scaled according to corresponding images taken after probe removal by formamide. Scale bars correspond to 10 µm. Representative images of four independent experiments are shown.

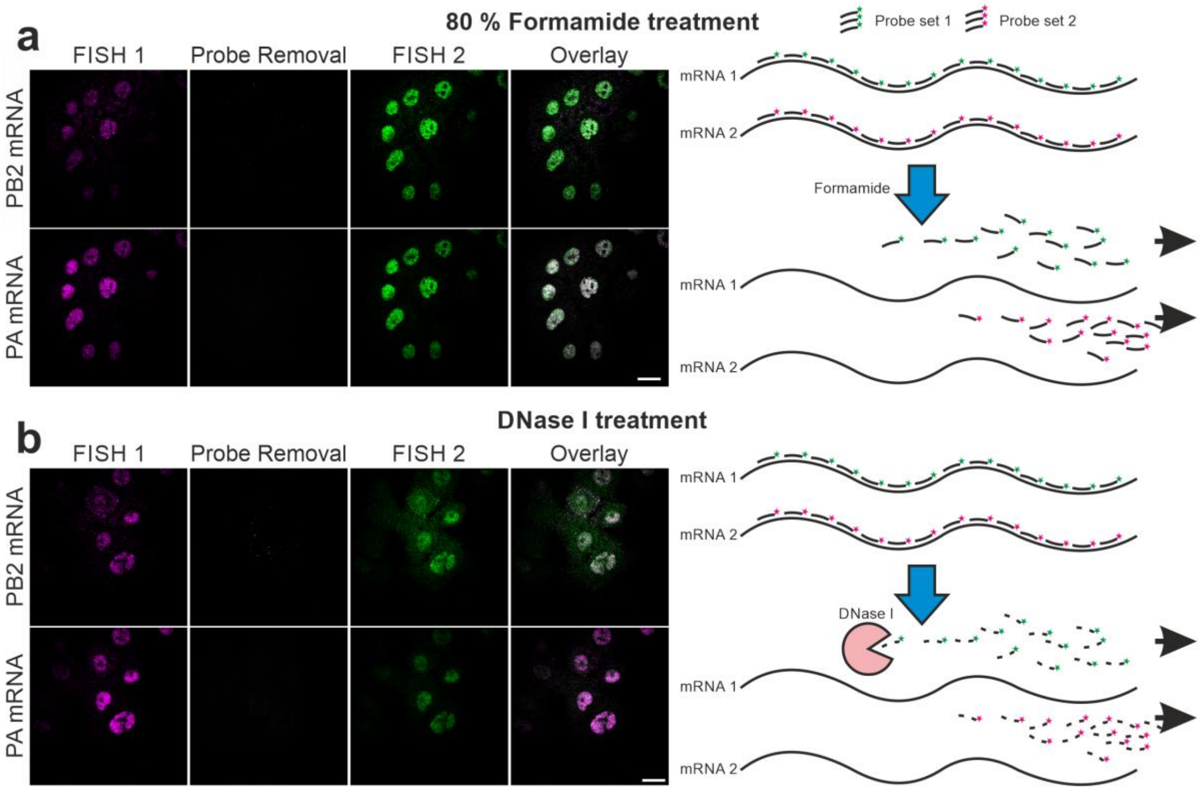

Supplementary Figure 2: Comparison of FISH probe removal by formamide and by DNase I probe digestion. A549 cells were infected with A/Panama for 10 h, fixated and stained by FISH targeting mRNAs of PB2 (Atto550-coupled probes) and PA (STAR635P-coupled probes). DNA-Probes were removed either by washing with 80 % formamide for 10 min at 37 °C (a) or by cleavage with DNase I for 3 h at 37 °C (b). FISH 1 corresponds to the first staining cycle. After treatment with formamide, the signal intensity of the following labelling cycle (FISH 2) was significantly increased using the same probe sets and fluorophores as in the first FISH cycle. This was not observed for probe removal by DNase I. Here, we found partially decreased signal intensity for FISH 2 in comparison to FISH 1. Note, for improved visual distinction FISH 1 and FISH 2 are shown in different colours (magenta, green) although the same fluorescent probe sets were used. Scale bars 10 µm. Experiment was performed once as proof of principle.

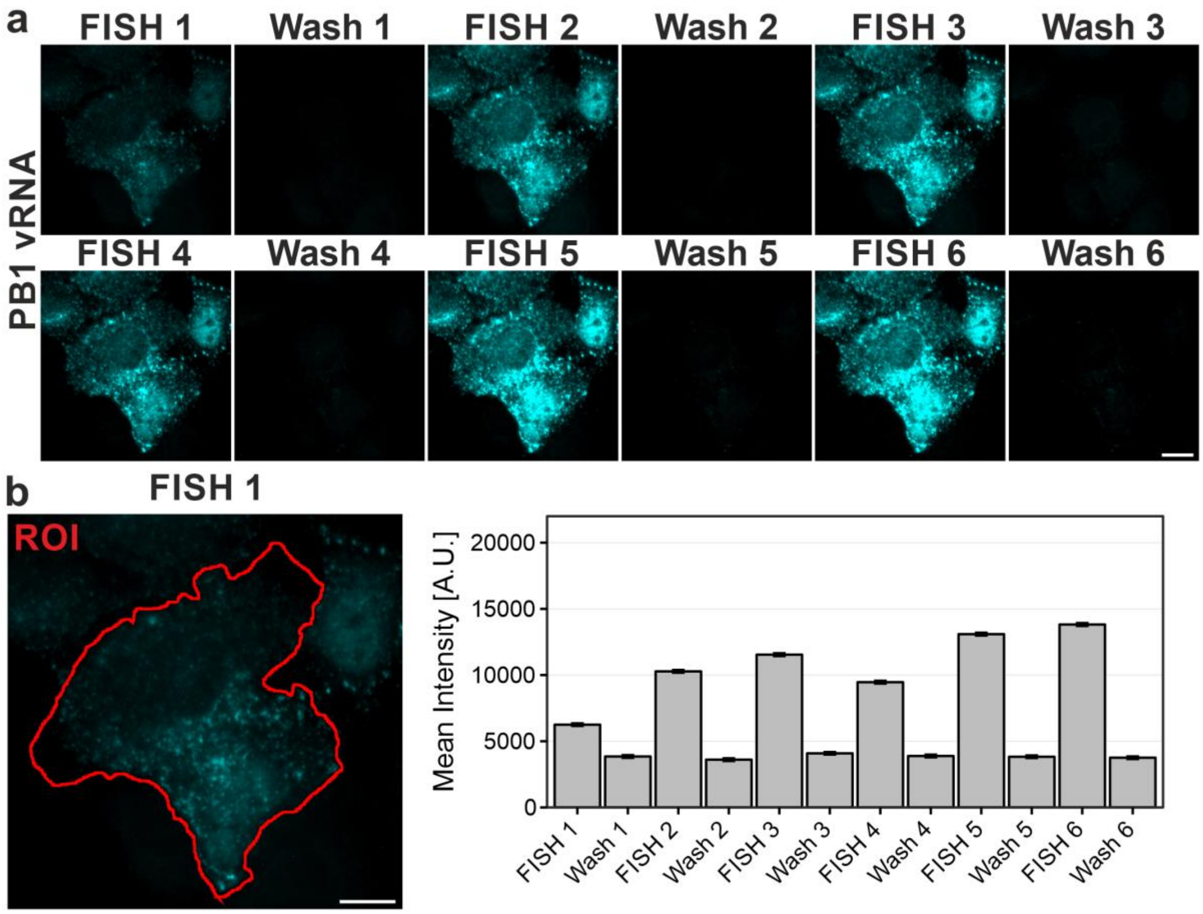

Supplementary Figure 3: Mean fluorescence intensity of PB1 vRNA in six runs of MuSeq-FISH. A549 cells were infected with A/Panama and fixated 10 h p.i. (a) FISH was performed to visualize PB1 vRNA using Atto550 probes in six sequential cycles. After each staining probes were removed by formamide washes. (b) A ROI was defined to measure the mean fluorescence intensity before and after removal of probes by ImageJ. The data documents successful PB1 vRNA staining and probe removal in each cycle. Moreover, formamide treatment enhanced FISH signal intensity of subsequent FISH staining - with the strongest increase after the first cycle. Specific FISH signal was significantly higher than the signal remaining after washing with formamide. For images, fluorescence intensity was scaled equally for all cycles according to that of images after formamide washing. Mean and s.e.m. of all pixels within the ROI are shown. Experiment was performed once as proof of principle. Scale bars correspond to 10 µm. Source data are provided as a Source Data file.

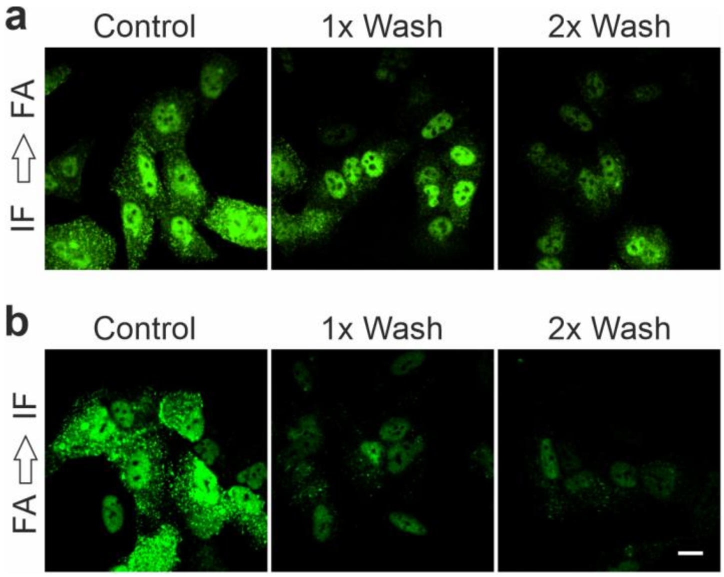

Supplementary Figure 4: Influence of formamide (FA) treatment on immunofluorescence (IF). A549 cells were infected with A/Panama and fixated 10 h p.i. IF for influenza NP was either conducted before (a: IF→ FA) or after (b: FA→ IF) washing with 80% formamide. As a respective intensity scaling control, antibody staining was conducted without formamide treatment. Irrespective of the particular order, in general, any formamide wash decreased IF signal intensity significantly. But the overall signal was still significantly higher when immunofluorescence was carried out first (upper row) rather than after a formamide wash (lower row). For this reason, immunofluorescence labelling is strongly recommended to be performed before the MuSeq-FISH cycles. Scale bar corresponds to 10 µm. Images are from one experiment as proof of principle.

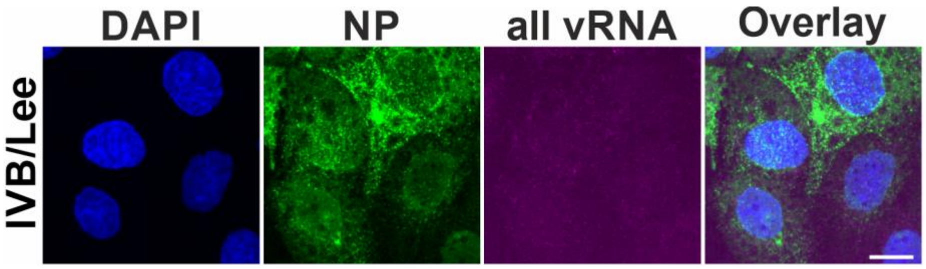

Supplementary Figure 5: Detection of vRNA in IVB/Lee infected A549 cells. Cells were stained by a mixture of all Atto550-coupled viral genomic RNA probe sets of A/Panama 10 h p.i. (MOI 5). Using the same conditions of image acquisition as for A/Panama, no signal of vRNA probes could be detected (see ‘all vRNA’). NP was stained by immunofluorescence using first an unlabelled primary mouse antibody (MCA403, Bio-Rad, Hercules, Germany) targeting IVB NP and second an Alexa Fluor 488-labelled goat anti mouse antibody (ab150117, Abcam, Cambridge, UK) . DAPI was used as counterstaining for nuclei. Scale bar corresponds to 10 µm. Images are from one experiment.

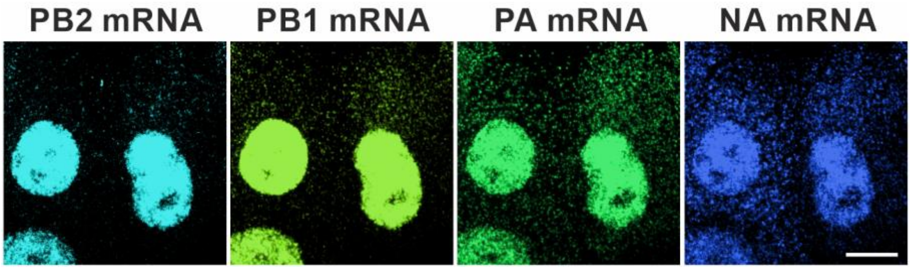

Supplementary Figure 6: High nuclear density of polymerase and NA mRNA species. Images correspond to mRNA data of Fig. 1. Signal intensity contrast was increased to demonstrate the occurrence of spot-like vmRNA signals in the cytosol. Scale bar corresponds to 10 µm.

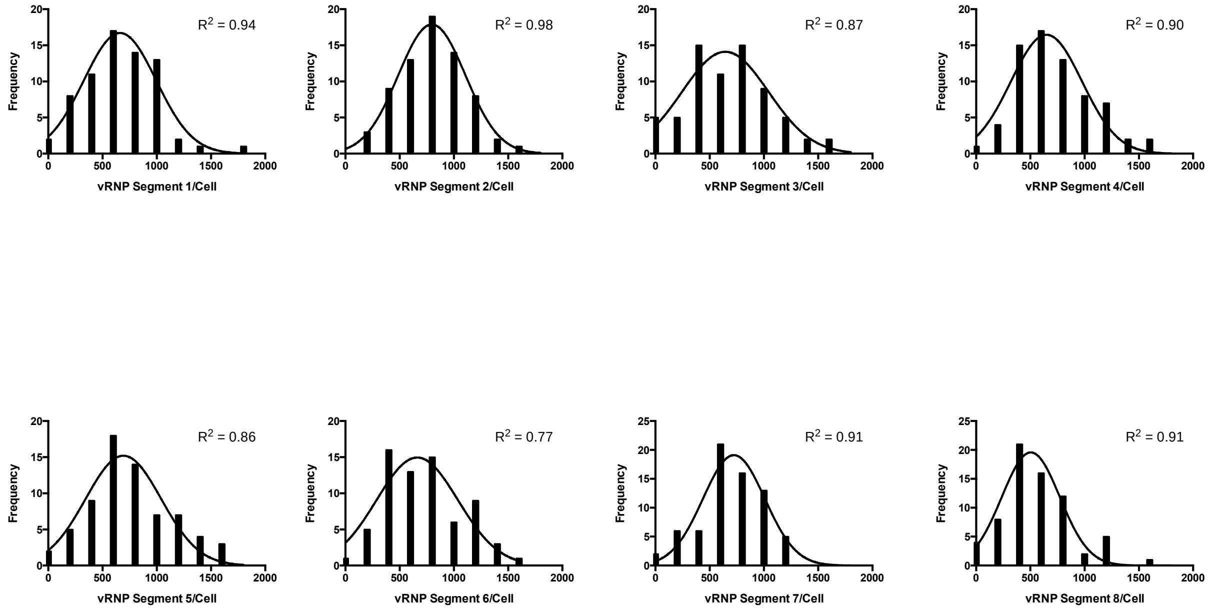

Supplementary Figure 7: Frequency distribution of the number of vRNP segments per cell for all eight vRNP segments. Data are taken from 69 cells (see Fig. 2). Distributions were fitted by a Gaussian function (straight line) using PRISM (version 6.0d, GRAPHPAD Software, Inc.). All fits passed normality test by D'Agostino-Pearson with p=0.05 (one-sided). R_2 is the correlation coefficient. Source data are provided as a Source Data file (see page Segment Distribution (data of Fig. 2)).

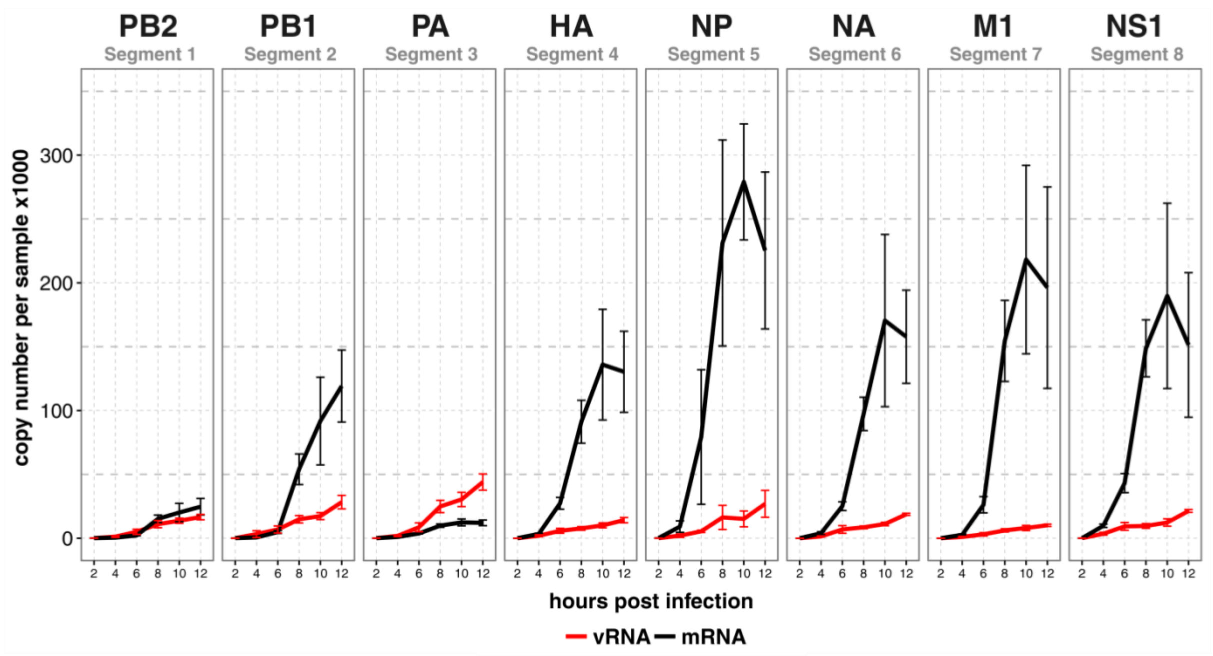

Supplementary Figure 8: qRT-PCR measurements of vRNAs and vmRNAs in A/Panama-infected A549 cells. A549 cells were infected with A/Panama at MOI of 5 for 2, 4, 6, 8, 10, and 12 h. Afterwards, RNA was extracted with an RNeasy extraction Kit (Qiagen, Hilden, Germany). The extracted mRNA was specifically transcribed with an anchored poly-dT-primer (Life Technologies, Henningsdorf, Germany) and the vRNA with an UNI12-primer (AGCAAAAGCAGG) recognizing the highly conserved 5'end of the viral segments into DNA by SuperScript IV polymerase (Life Technologies, Henningsdorf, Germany). Subsequently, remaining RNA molecules were cleaved by RNase H (Life Technologies, Henningsdorf, Germany) and qRT-PCR measurements were performed with SYBRgreen (KAPA Biosystems, Wilmington, MA, USA) and segment-specific primers. Three biological and three technical replicates were measured. The results show a strong increase over time for vmRNAs except for PB2 and PA mRNAs. This increase of vmRNAs is stronger compared to the raise of vRNAs. At 10 h p.i. the maximum values for HA, NP, NA, M1 and NS1 mRNAs were reached. Note, mRNA for M2 and NS2 were not measured (see Material and Methods). Genomic vRNAs increased continuously without reaching a plateau phase within the time frame of the experiments. Time dependence of expression levels were similar for all vRNAs. Mean and s.e.m. of $n = 3$ independent experiments are shown. Source data are provided as a Source Data file.

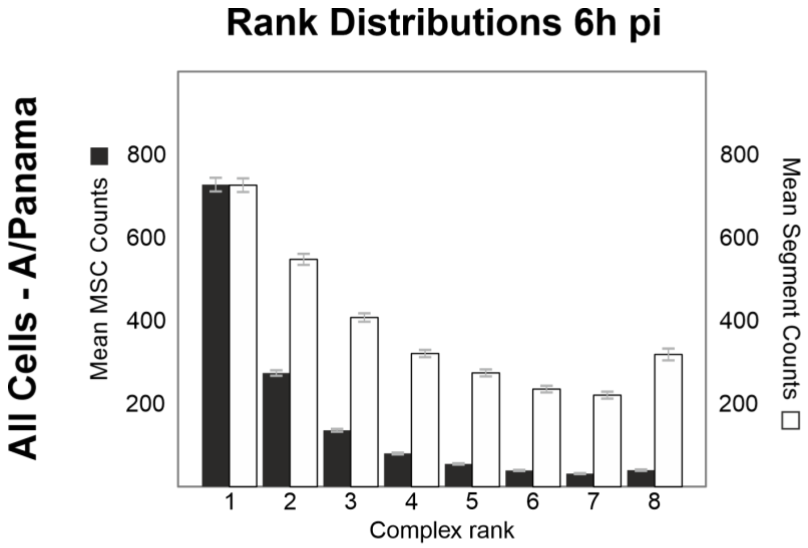

Supplementary Figure 9: **Frequency distribution of MSC ranks after 6 h p.i. (MOI 5).** A total of 2.6x10$_5$ distinct segment spots were detected in *n* = 84 A/Panama-infected A549 cells of three independent experiments and binned into 2.1x10$_4$ MSCs. Mean ± s.e.m. are shown. Source data are provided as a Source Data file.

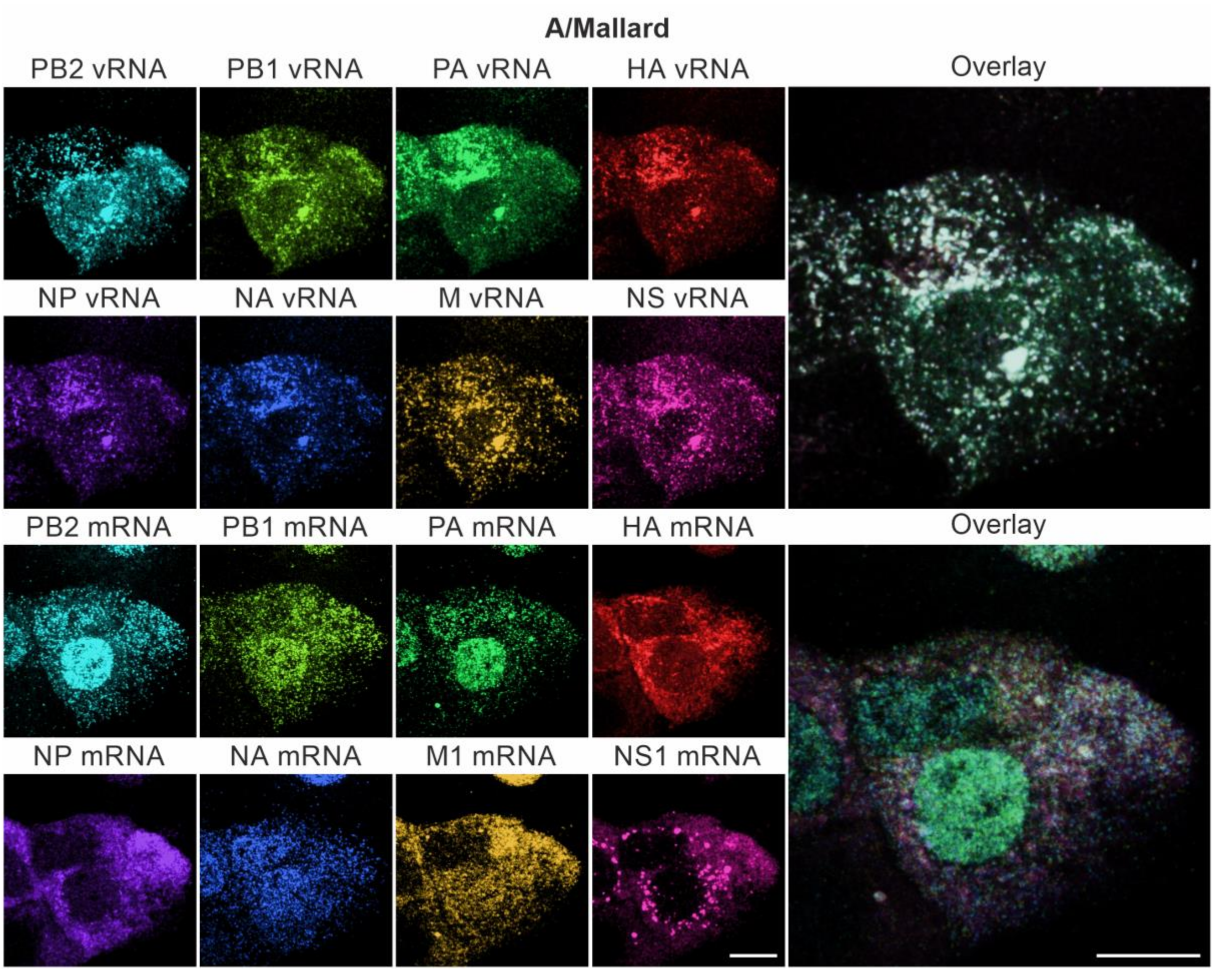

Supplementary Figure 10: Localization of vRNA and vmRNA in A/Mallard-infected A549 cells identified by MuSeq-FISH. Viral genomic RNAs were stained by FISH 10 h p.i. (MOI 5). To cover all vRNAs and major vmRNAs, 12 cycles of FISH labelling were performed. Along these cycles each vRNA was targeted twice, once with Atto550 (shown here) and once with STAR635P (Supplementary Figure 1) labelled probe sets (for details see text). High degree of colocalization was observed for all vRNA segments (white colouring in vRNA overlay). IAV NP stained by immunofluorescence displayed the same spot pattern as the vRNA spots. DAPI labelling was performed to exclude nuclear vRNA spots for further colocalization analysis. All major unspliced vmRNAs were stained. Images represent max-z-projections. The intensities of images were scaled according to corresponding images taken after probe removal by formamide. Scale bars correspond to 10 µm. Representative images of *n* = 3 independent experiments are shown.

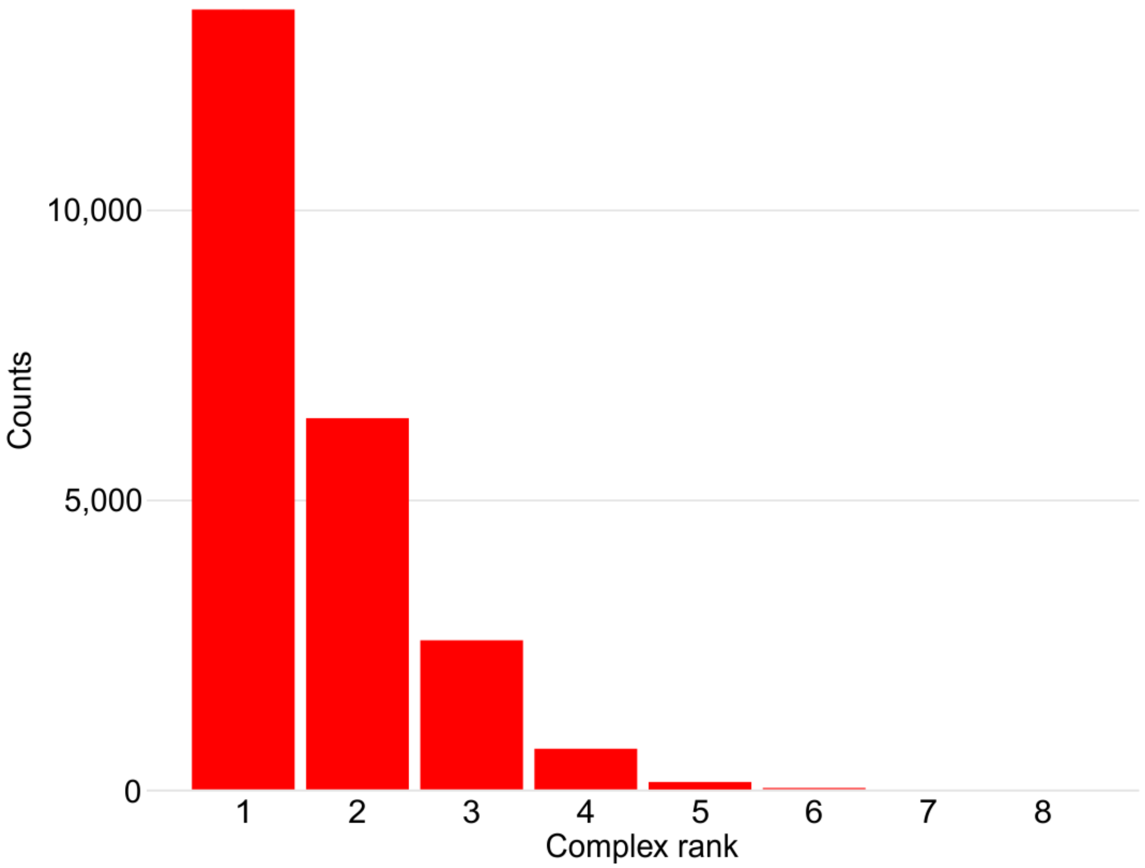

Supplementary Figure 11: Colocalization of the different A/Panama vmRNA species of a representative microscopy position. All vmRNAs were analysed with the same settings for identification of colocalization that were applied to vRNA. Source data are provided as a Source Data file. A representative position from data of Fig. 1 was used to analyze vmRNA colocalization.

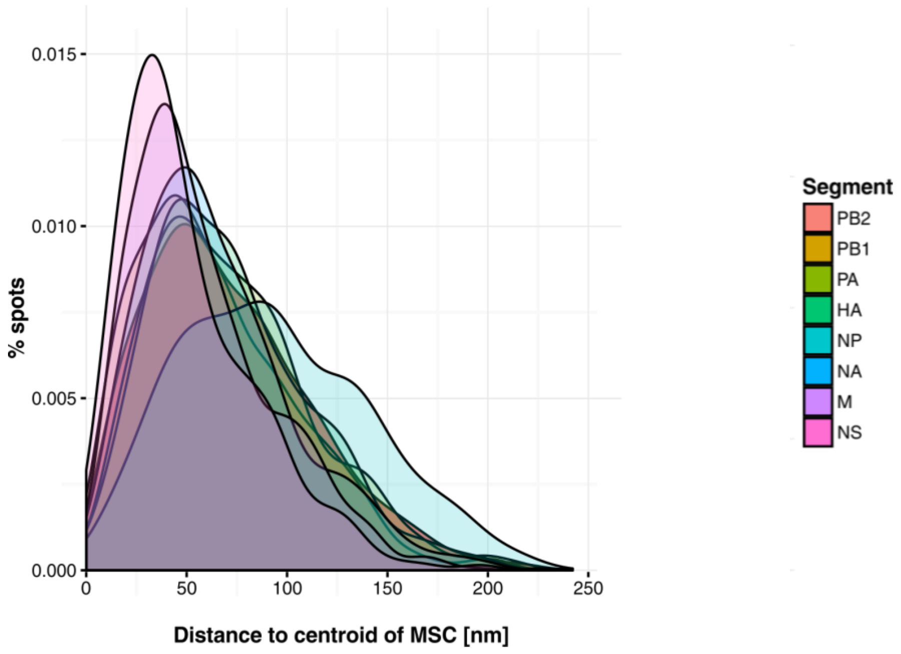

Supplementary Figure 12: Distance of vRNP spots to Center of Mass in MSCs. vRNA spots colocalising within a cylinder of radius 300 nm and height 1000 nm were binned into one MSC. For MSCs, the Center of Mass (centroid) in x and y directions were calculated. Then, distributions of distances of spots to centroids were calculated for each segment. High distances indicate sub-optimal image registration and a bias towards a shift of spots of this segment in comparison to the other segments. Here, segment 5 (NP) showed the least optimal registration as its distribution was shifted towards higher distances. However, all spots and their peaks are well localized within the colocalization radius of 300 nm.

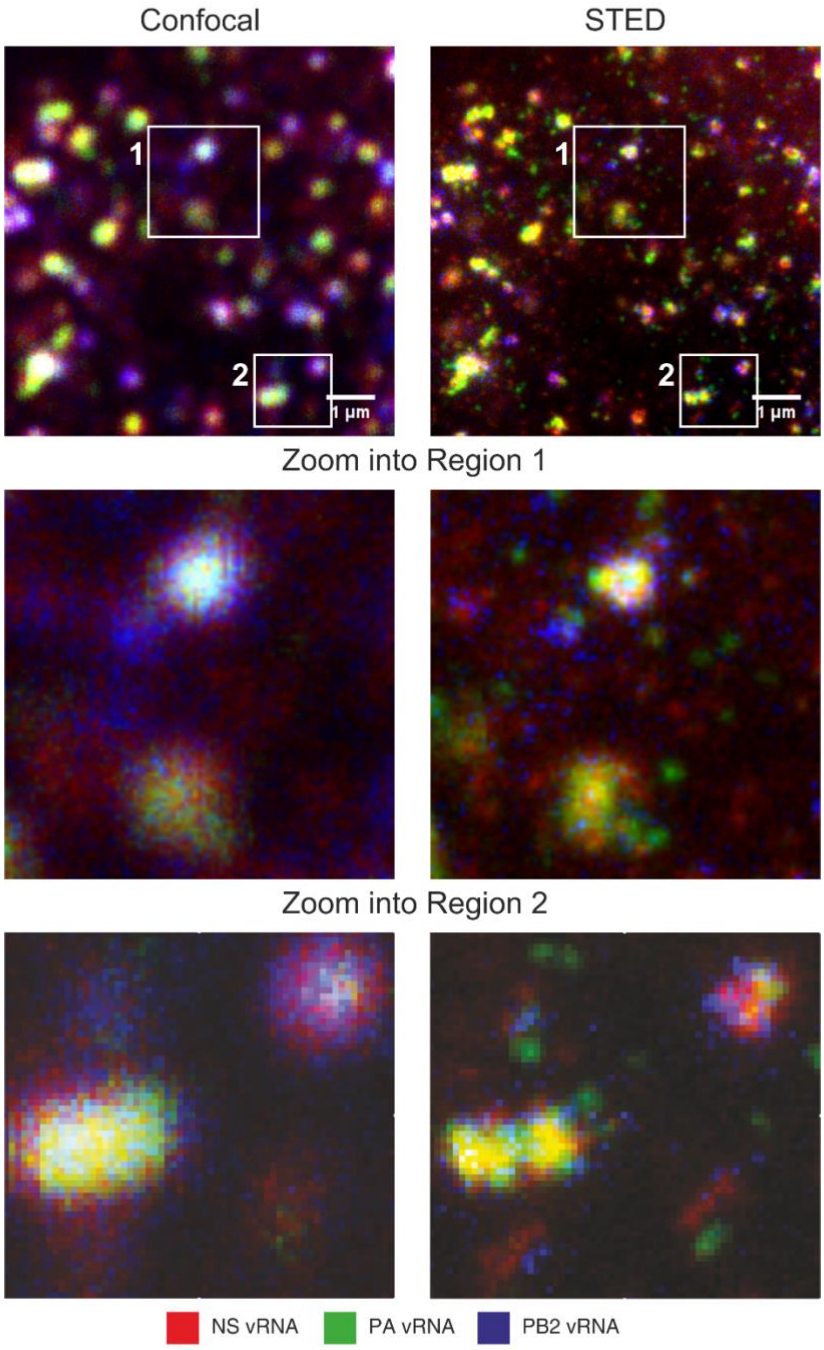

Supplementary Figure 13: STED vs. confocal microscopy. A549 cells were fixated at 10 h p.i. and FISH staining was performed with three colours: NS vRNA (red, STAR635P), PA vRNA (green, Atto594), PB2 vRNA (blue, Atto655). Images were taken in confocal as well as in STED mode. Lateral resolutions were calculated to be 310 nm (confocal) and 79 nm (STED), respectively. Images show high degree of colocalization for all three colours. Most of confocal single spots were also classified as single spots by STED (Region 1). In a few cases multiple spots identified by STED appear as a single spot in the confocal image (Region 2). Scale bars correspond to 1 µm. Representative images of n = 3 independent experiments are shown (for details, see Methods).

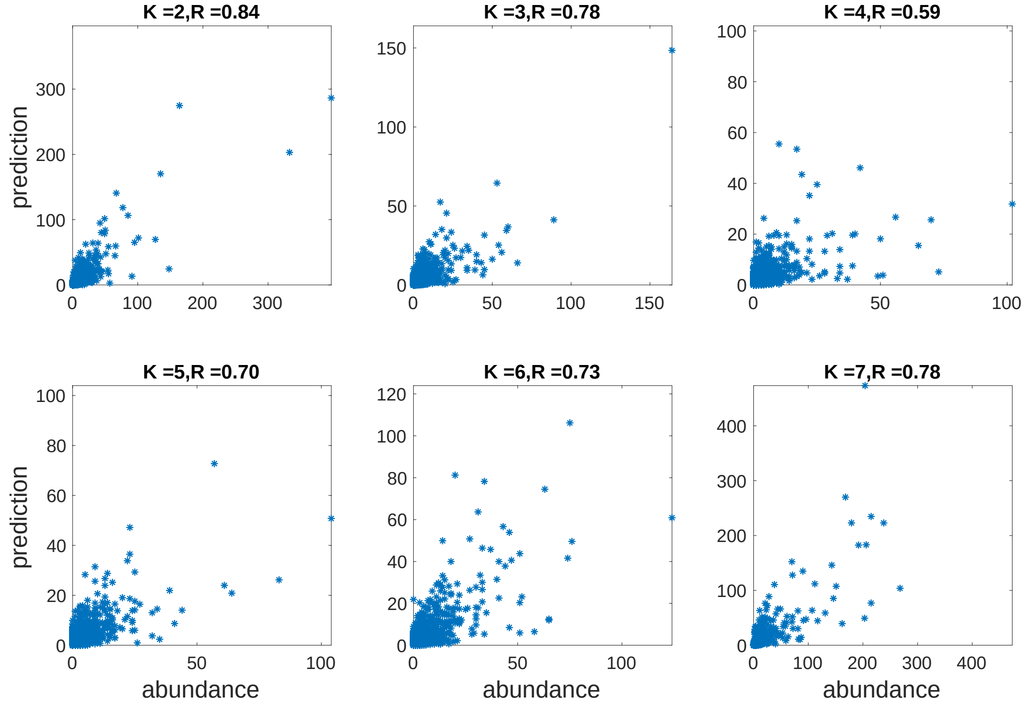

Supplementary Figure 14: **Predicted vs measured abundances for individual MSC ranks 2 to 7.** Prediction is based on the mathematical model described in the Method Mat. and Meth. section. The plot shows predicted vs. measured abundance of an MSC of specific rank and specific composition. The Pearson correlation coefficient (R) is shown for each plot. For an exemplary illustration see Supplementary Table 2. Source data are provided as a Source Data file.

Supplementary Table 1

A/Panama PB2 vRNA	A/Panama PB1 vRNA	A/Panama PA vRNA	A/Panama HA vRNA
AACGGGACTCTAGCATACTT	CGAGTCTGGACGGATTAAGA	CAATGCGTCTTGGTTCAACT	TTGCTGGGGTTCATCATGTG
TGTGCTAATTGGGCAAGGAG	CGGATTGATGCCAGAATTGA	CTTTGATCTTGGGGGGCTAT	CGAAGCATTAAACAACCGGT
CAGAAGATACGGACCAGCAT	GGAGACCGGTTGGAATTTCT	TCTTAGGGACAACCTCGAAC	CTGCATAGGGTCAATCAGAA
AAAGCACATCCGGAGTGGAG	GTGCTGCAACTTGTTCGAGA	AAGGATTTTCAGCGGAGTCA	TGCTGAGGATATGGGCAATG
CACTTTAATTGAAGACCCAG	GCCATAAAGAGATTGAGTCT	GGCTAAGTCGGTGTTCAATA	AGATCTCTGGTCGTACAACG
CAATTCTCGGAAAAGATGCC	TGGATGAGGATTATCGGGGA	CCAAGGGAGTGGAAGAAGGT	CCAAATCAACGGGAAACTGA
TCAACTACAACAAGACCACT	ACATTCCCGAAGTCTGCTTA	ATAAATCAGAAGCATGGCCC	AAAGCACTCAAGCAGCAATC
CTTGGGACATTTGATACCAC	CCCAATCAAAGGCAGGACTA	TTGTATGTGAGGACAAACGG	CACAGGACAAGCAGCAGATC
TCTCAGAATCCTGCAATGTT	CTAAAGAAGCTGTGGGATCA	CCAAATTTCAAGGCCTATGT	GTACGGTTTCAGGCATCAAA
GATTAACGGTCCTGAGTCGG	TCAGACGAGAAGATCATTCG	TACTGTGTCCTTGAGATAGG	TTGGGAGGGAATGGTGGACG
ACACAGGGAACAGAGAGACT	AATGACCTTGGACCAGCAAC	AGAGGGAAGGCGAAAAACCA	TCGCGGGTTTCATAGAAAAT
TGGTTAGCATTGATCGGTTT	CAGTTTTGGAGTGTCTGGAA	CATTAATACTGCCTTGCTCA	AACTAGAGGCATATTCGGCG
GTCAGCAAAATGGGTGTGGA	GGATAGATTCTACAGGACCT	GACATAAGCGATTTGAAGCA	GATGCGGAATGTACCAGAGA
GACAGTGTGATGGGAATGGT	TTTTGCCCTCATAGTGAATG	AGTAGACTTTGACAACTGCA	ACACTCTGAAATTGGCAACA
TGCATCAGCTTTTAAGGCAT	AGTACGGTTTTAGGAGTCTC	TCAGAGTTGCAGGACATTGA	TGTCCCAGATATGTTAAGCA
TTTCGTCAACAGGGCAAACC	GCATCGACCTGAAGTATTTC	ATTACCTGCTGTCATGGAAG	AGATGCACCCATTGGCAAAT
GTTTTCACAAGAGGATTGCA	GAGAATGAAGCTCCGAACAC	CAAACCACACGAAAAGGGGA	CTCCTCGGGGTTACTTCAAA
AGCCGAAGCAATAATCGTGG	AAGGATACATGTTCGAGAGT	CAAATTCCTCCTGATGGATG	GTAAAACCGGGAGACATACT
AGATTGGTTCAGCTCATAGT	GCATCGCACCAATAATGTTC	GAATGGACCTCCTTGTTATC	CTCCAGCAGAATAAGCATCT
CCGACTGAAGAACAAGCTGT	GGTTCGTGTACTTCGTTGAA	AGGAACTATGCGCAGGCTTG	CCAACAAACTGTAATCCCGA
GGTGGACATTCTTAGGCAGA	AACACCCGGGATGCAAATTA	TCAGTCCGAAAGAGGCGAAG	GAGTACGGACAGTGACCAAA
ATCAGCAGATCCACTAGCAT	CCAAAGATGCAGAGAGAGGT	CCAGGCTATTTACCATAAGA	CTGAACGTGACTATGCCGAA
GAGGAATGACGATGTTGACC	GCTAATGAATCGGGAAGGCT	GGAAAGCAGGGCTAGGATTA	TCGCTCAGAATGGAACAAGC
GACTCAAGGAACGTGTTGGG	GAAATCAACCGGCAGCAACT	CCACAAAGGCAGACTACACT	GAAAGCTTCAATTGGACTGG
GGCGGAACAAGCAGTATATA	GCCAGACTTATGATTGGACA	GTGACAAGGAGAGAAGTCCA	CGGCACACTGGAGTTTAACA
TAGAGAGAGAACTTGTCCGA	AGGGTGGACAAACTAACTCA	CCGAAGTTTCTACCAGATTT	CTTATGATGTGCCGGATTAT
GTCAAGATACGCCGAAGAGT	ACCTGAGGATAATGAGCCAA	CAACACTACTGGAGCTGAGA	GGAATGGGACCTTTTTGTTG
ATGGACCTGTGACAAGTACG	AATACAGAAACTGGGGCACC	GGCATGGACAGTAGTAAACA	GAATATGCGACAGTCCTCAC
GCTGTGACATGGTGGAATAG	CAGAAAAGGGGAAGTGGACG	TAGAGGGGAGAGACAGAACA	GTTCAGAGTTCCTCAACAGG
CAGAAATGGTTCCGGAGAGA	TTTTCCTAAAGGTTCCAGCG	CAAGGCGAATCAATAGTGGT	TTTCGCTCAAAAACTTCCCG

A/Panama NP vRNA	A/Panama NA vRNA	A/Panama M vRNA	A/Panama NS vRNA
GAAGGCAACGAACCCGATCG	ATTGTTGTGTTTTGTGGCAC	GAGTACCTGAGTCTATGAGG	TTCATGCAAGCATTACAGCT
GAGTTTTCGAGCTCTCAGAC	AAGTCTGGTGGACCTCAAAC	GAAAAGAGGGCCTTCTACGG	AATGGCGAGAACAGCTAGGT
TCACTGGAAATACGGAGGGA	GTGCTTTTATGTGGAGTTGA	ATCGACTCTTCAAACACGGC	TCCACTTACTCCAAAACAGA
CCAAATCAGTGTGCAACCTA	GAAAGAGGTAATATGTCCGG	CTTGCACTTGATATTGTGGA	TCTACAGAGATTCGCTTGGA
TACTAATCAACAGAGGGCCT	GCAGATAAATAGGCAAGTCA	TGTTGCTGCGAGTATCATTG	ACACAGTTCGAGTCTCTAAA
ACTGAGAAGCGGGTACTGGG	GGTCCAAACCTAACTCCAAA	TGCAACGATTCAAGTGACCC	CGGAGGGCTTGAATGGAATG
ATATGGGATCAAGTACTCTT	AGAAGTCACGCTCAGGTTAT	CAGAAACGAATGGGGGTGCA	AAAATGCAATTGGGGTCCTC
GCGGGGGAAACTTTCAACTA	CGTGTGGATGGGAAGAACGA	TCCAGTACTGGTCTAAGAGA	GGACATACTATTGAGGATGT
GTTAAGCTTCATCAGAGGGA	TGGGCCTTTGATGATGGAAA	AACCGTTGGGACTCATCCTA	AGGGAGCAGTTGTTGGCGAA
TCAGACCGAACGAGAATCCA	AGGGGGTCATGGAGTGAAAG	AGGCAAATGGTGCAGGCAAT	TTACTAAGGGCTTTCACCGA
AATAGCCAAGTATACAGCCT	GCCTGGATCCTAACAATGAA	CATGGAGATTGCTAGTCAGG	TGGCCGACTAGAGACCATAG
TGTGTATGGACCTGCAGTAT	TATGTGTGCTCAGGACTTGT	CACTACAGCTAAGGCTATGG	GCGAATTTCAGTGTGATCTT
CACAAATCTTGCCTACCTGC	GGATTATAGCATTGTTTCCA	GTCTCATAGGCAAATGGTGG	TCAGAATGGACCAGGCAATC
TGAGAGAAAGTCGGAACCCA	CTCCTGTTATCCTCGATATC	CTGGTATGTGCAACATGTGA	AACTGGTTCATGCTAATGCC
GCACAAAGAGCAATGGTGGA	CGTTCATACTAGCAAATTGT	TAACCACCGAAGTGGCATTT	CTGCTTCGCGATACATAACT
GGGCGGAAAACAAGAAGTGC	ACTTTTCATTGAGGAGGGGA	GGGCCTCATATACAATAGGA	TAAAATGACCATGGCCTCCA
CGAAATTTCTGGAGAGGTGA	TCGGAATGCGTTTGTATCAA	ATTTTGGGGTTTGTGTTCAC	AACACTCTCGGTCTAGACAT
TCAGAATGGTCAAACGGGGG	ATAGTATTGGTTCATGGTCC	CTTGAGGCTCTCATGGAATG	CGAGATCAGAGGTCCCTAAG
GAATGTGCTCTCTGATGCAG	ATTTACGATGGGAGACTTGT	AAAGCCGAAATCGCGCAGAG	AACTGAGTGATGCCCCATTC
CGACAGCTGGTTTAACTCAC	TGAAAATGCAACTGCTAGCT	CGTATGTTCTCTCTATCGTT	TCCGGAAGCAAGTTGTAGAC
CCCATATACAGGAGAGTAGA	CACGATGGAAAAGCATGGCT	AAGATGAGCCTTCTAACCGA	GGATTCCAACACTGTGTCAA
GATATCTGGAAGAACACCCC	TAGCATGGTCCAGCTCAAGT		AGCAGGGTGACAAAGACATA
AGAAAATGGTGCTCTCTGCT	CTTATCGAACCCTATTGATG		
TGATCCAAAACAGCTTGACA	TGGACAGGGAACAACACTAA		
CTCAGTGATTATGAAGGGCG	ACAAGTGTTATCAATTTGCC		
CCAAATGTGCACTGAACTTA	AACCTTATGTGTCATGCGAT		
GCCAGAATGCAACTGAGATT	AGGATTTGCACCTTTTTCTA		
AGGCACCAAACGGTCTTATG	ACAACCAAGTAATGCTGTGT		
ACATCAAAATCATGGCGTCC	CTACTGTAACATTGCATTTC		
GCAGGGTTAATAATCACTCA	CTTATGCAAATAGCCATCCT		

A/Panama PB2 mRNA	A/Panama PB1 mRNA	A/Panama PA mRNA	A/Panama HA mRNA
ATTTCTCTCCGGAACCATTT	TGTGTTCTGTTGACTGTGTC	GATCATCAAGTTCTACCACT	GTGATTGTTTTCACTAGCGT
CTCCATAGAGTTTGTCCTTG	CAGTTTCTGTATTTGTCGTC	TACTACTGTCCATGCCATTG	CGCATATTCTACCTGTTGAG
TGGACCGTACTTGTCACAGG	TGGGATTCTTCAAGGAAGGC	TCCAGTAGTGTTGCAGATAC	TCAAGGATTTGGTGAGGACT
ATGAACAGGGCCAAAGGTTC	GGCATGAGCTCTCAAAGATC	GTCACTCCAATTTCGATGAA	TCCTTATTTTGGAAGCCATC
GGCGTATCTTGACTTGATTT	TGTTTGTTGAACGACTTCCA	TAGCCTGGTCTTAATCCTAG	GCGTTCAACAAAAAGGTCCC
TGTATGCAACCATCAAGGGA	CTTGAGTTAGTTTGTCCACC	GTTCGAATCCATCCACATAG	AACAGTTGCTGTAGGCTTTG
TCGGACAAGTTCTCTCTCTA	GTCCAATCATAAGTCTGGCG	AAGGAGGTCCATTCGGAAGT	TAATCCGGCACATCATAAGG
GCAACTGGGAGAAATCTTGT	TGCCGGTTGATTTCTGTTTA	AGGAATTTGGACCGCTGATA	ATGAGGCAACTAGTGACCTA
TCAACATCGTCATTCCTCAC	TAATTTGCATCCCGGGTGTT	GATCGCATCATATAGTGGGA	TTGTTAAACTCCAGTGTGCC
GGCTGCAATAATTAGGCTTT	TCAACGAAGTACACGAACCC	CTTGGAATCTTCTCCTCATT	AGAGCTTGTTCCATTCTGAG
CGGCTCTTCTTACTATGTTC	TTTGGCCTTCTTTTCATTAC	AGAGCCCACTTTAGTTGACT	TTTCATTGTTCGGCATAGTC
ACAGCTTGTTCTTCAGTCGG	TCTTTCTCACAACATTTGCC	TGCAGTTGTCAAAGTCTACT	TGATTTGGTCACTGTCCGTA
CTCTCTTTTGACTGATGACC	GCTCTGTGTCTTGTGAATTA	TGCTTCAAATCGCTTATGTC	CCTGATGCTTGAGCATATAT
TGTTTGGAGATTGCCTGTAA	TCCCCAGTGATTGTGAAAGA	TTCAGGTTCGTCACTATCAT	TCGGGATTACAGTTTGTTGG
ACCATTGTGAACTCCTCATA	TCATCGCCAAGAACATTCGA	GCTCTATCCAGATTGAATCA	CCGGTTTTACTATTGTCCAA
TATGAGCTGAACCAATCTCC	ATTATTGGTGCGATGCTCAG	GACACCTCTGCTGTGAAATA	TAACCCCGAGGAGCAATTAG
TATTGACTGTTCGTCCCTTC	TCGAACATGTATCCTTTTCC	GTACTCAGTGGCTCTACAAT	GCATCTGACCTCATTATTGA
GTCACCTCTAACTGCTTTTA	TTCGGAGCTTCATTCTCTTA	GTTGAAAATCGTCCATTGCT	TGTTGCCAATTTCAGAGTGT
CACTTTCGCATCTTTCTGAA	TAGCATTTCTGCGGGTATTT	AAATTGGTTTTTCGCCTTCC	TCTCTGGTACATTCCGCATC
GTCATATCTGGTAATACTCC	AATACTTCAGGTCGATGCTT	CTTCCTTTTATGATGAATCC	CCGAATATGCCTCTAGTTTG
CATTGACATCTCTGTGCTTG	GAGACTCCTAAAACCGTACT	CCTATCTCAAGGACACAGTA	CATTTTCTATGAAACCCGCG
CGATCAATGCTAACCACCAC	GACTTCTTTTTGCTCATGTT	CATAGGCCTTGAAATTTGGC	CTGAAACCGTACCAACCGTC
GTTAATCTCCCACATCATTG	CCACAAATCCATAGCGATAA	CGTTTGTCCTCACATACAAG	TGTGCCCTCAGAATTTTGAT
TGACCAAAACCGACTCAGGA	TCATTTATTCCAGACACTCC	TCTCTATCTGCTGGAGTGAC	ATTTGGTTGATTGCTGCTTG
GGATTCTGAGACCATTGGAT	GGTCCAAGGTCATTGTTTAT	GAGGACTCGGCTTCAATCAT	GGAATTTCTCGTTCGTTTTC
AGTGGTCTTGTTGTAGTTGA	TCTGATACCAATAGTCCTGC	ACAGGCTATTGAACACCGAC	ATTTCTCGAGGTCCTGAATT
AATCCTCTCAAGACAGCGGA	GTTGTATAAGTTTGGTCCTC	CCTTCTAATTGTGGAGATGC	GATCTATTTTAGTGTCCTCA
AGGTTACTCAGTTCATTGAT	GCAGACTTCGGGAATGTGAA	TTTTCTTGACTCCGCTGAAA	TCAGCATTTTCCCTCAGTTG
TTGCCCAATTAGCACATTAG	AGACTCAATCTCTTTATGGC	GAGCCTGAACAACAAGGAGC	AGTTCCATTTCTGATTGACC
TTCGTTTCATTACCAACACC	TTCTGGTACATCTGTTCATC	AAGATCAAAGGTCCCAGGTT	TCAGCTCAACACCTTTGATC

A/Panama NP mRNA	A/Panama NA mRNA	A/Panama M mRNA	A/Panama NS mRNA
TCAGTTTCCATCTGTTCATA	ATGTTACAGTAGTTACCAGG	AAGTCTCTGCGCGATTTCGG	TCTTGGTCTACAACTTGCTT
CCTAATCTCAGTTGCATTCT	GTTGCATTCATATTGCTTGA	TTTTCCCAGCAAAGACATCT	GAAGCCGATCAAGGAATGGG
CATCAATCATCTTCCCGACG	CTATTATTGTTGGTTCACAC	ATGAGAGCCTCAAGATCTGT	CTTAGGGACCTCTGATCTCG
TGGATGTAGAATCGCCCAAT	TATGGTGGTGTTGGTCAGAT	CCTTAGTCAGAGGTGACAGG	TGATGTCTAGACCGAGAGTG
CTGAGTTTAAGTTCAGTGCA	CAGCGGAAAGCCGAATTGAA	GCGTGAACACAAACCCCAAA	CTTCCTTCAGAATCTTTTCT
AAGCTGTTTTGGATCAACCG	TCAGGATCGCATGACACATA	CTCCCTCTTAAGTTTTCTAT	GTCATTTTAAGTGCCTCATC
TGGGGTGTTCTTCCAGATAT	AATGCCTGTTGTTTAGTGTT	AAATGCCACTTCGGTGGTTA	TATCGCGAAGCAGGTGTGGA
ATCTACTCTCCTGTATATGG	CATTCATCAATAGGGTTCGA	TCACATGTTGCACATACCAG	CCTCAATAGTCATGTCAGTT
TAAAGGACGAGTTCCCTCAT	CCCAAATGAAATGGAACACC	CACCATTTGCCTATGAGACC	ATGAACCAGTTTCTTGACAA
TGAGTTAAACCAGCTGTCGC	TGCTATACACACTTGCTTGG	TAATGGATTGGTTGTTGCCA	CACTTTTTGCTTGGGCATTA
CGGTTCGAACAAGAGCTCTT	ATCGTGACAACTTGAGCTGG	AGCTGTAGTGCTGGCCAAAA	ATACTATGGTCTCTAGTCGG
CCTCTCCAGAAATTTCGATC	CGTAAATGAAGCTAGCAGTT	CTGACTAGCAATCTCCATGG	TCTTCGGTGAAAGCCCTTAG
TAAGCACTTCTTGTTTTCCG	ATACTATCTACAAGTCTCCC	TAGGATGAGTCCCAACGGTT	GTGAGATTTCGCCAACAACT
CGACTTTCTCTCACTTGATC	CTGAGGATTTTTTTGGACCA	TCATCTCTTAGACCAGTACT	
GATCTTCGATCTCAGCATTT	CAAACGCATTCCGACTCCTG	CATTCGTTTCTGATAGGTCT	
TATTAATGCAGATCTTGCCA	TACTGTACAAGTTCCATTGA		
AGATTTGTGAGCAACTGACC	TGAGCACTTCCTGACAATTT		
CATACACACAGGCAGGTAGG	ATAACAGGAGCACTCCTCGA		
TTCGGTCTGATTAGGCTGTA	ATCTGACACCAGGATATCGA		
TCCCTCTGATGAAGCTTAAC	TTATATCTACGATGGGCCTA		
CGCTTCTCAGTTCAAGAGTA	AACAAGTCCTGAGCACACAT		
AACGTAGGTTGCACACTGAT	AATGGCTACTGCTGGAGCTG		
CCATGACAGTTGACTTTTCA	CTTCTTCATTGTTAGGATCC		
CTCCGTATTTCCAGTGAATG	TTTGGAGTTAGGTTTGGACC		
TCTCGTCTGAGAGCTCGAAA	TGACTTGCCTATTTATCTGC		
AAAAGAGGGCACGATCGGGT	CGGACATATTACCTCTTTCA		
AATTGTCGTACTCTTCTGCA	ACTCCACATAAAAGCACCGA		
	AATACTGTTTGAGGTCCACC		
	CTGAGGTGCCACAAAACACA		
	CATGAGCCTGTTCCATATGT		

**A/Mallard PB2 vRNA**	**A/Mallard PB1 vRNA**	**A/Mallard PA vRNA**	**A/Mallard HA vRNA**
GTGTTGATAGGGCAAGGAGA	ACAGAAGGCCAGTTGGAATT	GGGGTTTTCAGCTGAATCAA	CGTCCTATTACGAATTCCAG
TGCGAAAGGGGAGAAGGCTA	TACAGATGCCACAGAGGTGA	CCCTTCAACAAATTGAGAGC	TAGGAACGGGACTTATGACC
GAGTGGAATCTGCGGTATTA	GCTCTTCAGCTATTCATCAA	AAATGAGGCGATGCCTTCTT	GTCTTATAATGCGGAACTCC
AAGATGCAGGTGCTTTGACA	CTGGGATTAACGAATCGGCT	CTCCTACGGACTGCAATAGG	GGAGGATTCAGAACCTTGAG
CAACCAAGAGGCTCACAGTC	GGATTTGTAGCCAACTTCAG	GTGTTCTCGAGATAGGAGAC	TATGTGAAGCAGAACACCCT
GGGGTTCAGGAATGAGGATA	TGGTTGGGATCAATATGAGC	CGGAAGACTAATCTGTATGG	GATCACATACGGAGCATGTC
TCTTCTCTGACTGTGAACGT	ATGAGAATCAGAATCCTCGG	GGAGTGTACATAAACACAGC	CACCTATTGACACCTGTATT
CACCGGAACAGAGTAGGATG	CCGTTGGAGGGAATGAGAAA	TTGCAGGGCTACTGAATACA	GGACGTACTGGTGATCAATA
ATAAAGCTCCTACCATTTGC	ATTAGCGAGGAGCATCTGTG	ATTTCACAGCGGAAGTATCC	GCAGACTATAATCCCTAACA
AGGCCAGTATAGTGGATTTG	AAGTTGAAGAGGCGGGCAAT	GCACATTGCGAGTATGAGAA	ATGTTCAAGCCTCAGGAAGA
AAGATGGAGTTTGAGCCCTT	CATTGAATAGGAACCAGCCG	GGGAAGACATTGCTCCAATT	TATACATCTGGGGAGTTCAC
CTGTGAAGATTCAGTGGTCC	GGATAAACTGACCCAAGGTC	AGTTGGATTGAGCTTGATGA	GTTTCACTTGGGCAGGAGTG
AAATGGTCCGGAATCAGTGC	ACCATTGCCTGAGGATAACG	GGCATGTGAATTGACAGATT	ACATTGGAGTTCATCACTGA
CATCGTCTATGATGTGGGAG	AGCATGGAGTATGATGCTGT	CTGGATCCAGAGTGAATTCA	TACCAGACTATGCATCCCTT
GGGAACAGAGAAGCTGACGA	GTTCAAAGGCAGGACTGTTG	CAGAGTCTAGATCGCTAGCA	GACCATTACAGATGATCAGA
CAGAGGGGAAACGTGCTCTT	AAGAAGCTGTGGGAGCAGAC	AGACAGTATGACAGTGATGA	CAGTGCCGAATGGGACAATA
ACTGAGAGAGTGGTCGTGAG	AACGATCTTGGACCAGCAAC	GGACTGCAAAGATGTTAGCG	CCTTTTAGGGAGCGACAATA
GAGGAGTGAGAGTCAGTAAA	CCCCGATAATGTTCTCAAAC	AGGGCATAAACCCCAATTAC	GACCATTATCGCTTTAAGCT
GATCGGAATATTGCCTGACA	ACCAACCTGAATGGTTTAGA	GAAGACATTTTTCGGCTGGA	AGAATTCGCCCTTAAGTTGG
GACAAGCAGCGTGTATATCG	ACTGGAGACAACACCAAGTG	GTATACCGCTATATGATGCA	CTGAAGACATGGGCAATGGT
GAGCATCAACGAATTGAGCA	GGTTCGTGTACTTTGTCGAA	TGCTCTCAACGGTCGAAGTT	AGACCAGAAGGCAACTGAGG
GATGTGTTGGGGACATTTGA	GAGCACTGACACTGAACACA	CTCTCAAGCTACCTGATGGG	AAGGAATTCTCCGAGGTAGA
AGACCCTACAATGTTATACA	TCTTCAGATCGAACGGTCTA	CCATTTCTGAAGACAACACC	ACCTTAAAAGCACTCAGGCA
CCCAGTACAGAGATGTCACT	TGCTTTGGCCAACACTATAG	AGAAGTGAACGCCCGAATTG	ACAGTCTCTACCAGGAGAAG
AGACGAGCAGTCAATCGCTG	CCGAGCGGATATGCACAAAC	TGAGGGCAAGCTTTCTCAAA	CCAGTGTTGAACGTGACTAT
GTGTTGCATTTGACTCAAGG	ACAAACACCGAGACTGGAGC	GTGGATGGATTCGAACCGAA	AGGTGACTAATGCTACCGAG
CGGGACTCTAGCATACTTAC	CTCAGAGAAAGGGAAGTGGA	GCCTTGAAAACTTTAGAGCC	TTTTGCTGGGGTTCATTATG
CCCTGTGTTCAACTATAACA	CAGCCATGGAACAGGAACAG	AAAGTCTCCCACCGAACTTC	ATATGCAACAATCCTCACAG
AGAACGCTATTCCAGCAGAT	ACTACATTCCCTTATACTGG	AAATCACAGGAACCATGCGC	GGAGCAAAAGCAGGGGATAT
CTCCTGAAGAGGTTAGTGAA	AAAGTGCCAGCGCAAAATGC	CGAGAGAGGCGAAGAGACAA	

**A/Mallard NP vRNA**	**A/Mallard NA vRNA**	**A/Mallard M vRNA**	**A/Mallard NS vRNA**
GGATGATGGAAAGTGCCAGA	GTATCGTCGTATTTTGTGGA	TGTCAACATAGAGCTGGAGT	AGAACTTTCTCGTTTCAGCT
GCAGAACATCTGACATGAGG	TAAGAGGAAGGCCACAGGAG	TGGATGTTGACGATGGTCAT	CTATTGCTTGAAGTAGAGCA
GCATTTACAGGGAACACTGA	CCATGGCTAATTCCAAATCA	TATCGGCAGGAACAGCAGAG	TACGTTTATGCAAGCCTTAC
CGAAAGGGCGACCATTATGG	TTCAGGGTCATTAGTGGTTG	GCCTGAGTCTATGAGGGAAG	ACAGAGAACAGCTTCGAACA
CAGTACAGAGAAATCTTCCC	GCCTTTGACTATGGAGATGA	AAAGAGGGCCTTCTACGGAA	ATGGCTGATTGAAGAGGTGC
AGAGCATCTGCAGGACAAAT	TGATAGCTCTAGCAACAGCA	CGTCGCCTTAAATACGGTTT	TGGCGAGAACAATTGAGTCA
TGGAGGAAACACCAACCAGC	GTATGGCAGACTATAGCATT	TTGTGGATTCTTGATCGTCT	CCTCCAAAGCAGAAACGGAA
ATTGGGCTATAAGAACCAGG	ATCCAGACGTTAGATGTGTT	AGTATCATTGGGATCTTGCA	GTAATGAGGATGGGAGACCT
AACTATCCACCAGAGGAGTC	TTGTTCATGTCAGCCCATTG	GGATGGGAGTGCAAATGCAG	TCTACAGAGATTCGCTTGGA
GACAAGAGTGGTCCCAAGAG	ACTATTCATTAGAGAGGGGA	TGAAAATTTGCAGGCCTACC	AACACAGTTCGAGTCTCTGA
GAGTATCAAGTTTCATCAGA	CTCAGAACTCAGGAATCAGA	TGCCGGTCTGAAAGATGATC	AAAATGCAATTGGGGTCCTC
CATTCTGCAGCGTTTGAAGA	TATGATGGGATGCTTGTTGA	CAATTGGAACTCACCCTAGC	TCCAGGACATACTGATGAGG
GAGCCAATTGGTATGGATGG	ACGAATTAGGCGTTCCATTT	CAGATGGTGCAGGCGATGAG	AAATCTCACCGTTACCTTCT
AAAACAGCCAGGTCTTCAGT	TCCTCATCGAACTCTTTTGA	CATGGAGGTTGCTAGTCAGG	GAAGAAGGAGCAATTGTGGG
ATAGATCCTTTTCGTCTTCT	CACTCGAATGGCACAATACA	TACCACCAACCCACTAATTA	CCCTAATACTACTTAGAGCT
AGAAGGATACTCTCTGGTCG	AGGGAACCACGCTGGATAAC	GATTGCTGATTCACAGCATC	TCAGTGTGATTTTTGACCGG
GCCAGTGGATATGACTTTGA	GCCCCGATAAATGTTATCAA	GAACGGTGACCACAGAAGTG	CTTTGCATCAGAATGGACCA
GAGGATCAGTGGCCCATAAG	GTTTGTCCCAAAATGGTAGA	AGAAGTGGCTTTTGGCCTAG	GTCAAAAATGCAATTGGGGT
AATCCTGGGAATGCTGAAAT	TGCCATGTGAACCAATCATA	AACAGGATGGGAACGGTGAC	ATCAGAATGGACCAGGCAAT
GGATCAAGTACGAGAAAGCA	AAAATGAGTGCAATACCCCC	ACAGGGCAGTCAAACTGTAC	ATGTCAAGGGACTGGTTCAT
TCGGATGATAAAGCGAGGGA	TTGCAACAGTATGTTTCCTC	TGGGAATGGAGACCCAAACA	GCTACCTAACTGACATGACT
CGGGACGATGGTGATGGAAC	TAGAGTATGGTGGACCTCAA	TTAGGATTTGTGTTCACGCT	AATGACTATTGCTTCAGTGC
CTCTGATGCAAGGATCAACC	GGGATCCTAATAATGAGAGA	CTGTCACCTCTGACTAAGGG	GGAAGAATCTGATGAGGCAC
ACTGGTATGGACCCAAGAAT	GAATGTTCCTGTTATCCTCG	CTCATGGAATGGCTAAAGAC	GCAAATAGTGGAGCGGATTC
CAACCTGAATGATGCCACAT	TGTCAGATTACAGGGTTTGC	GAAGAACACCGATCTCGAGG	GACATCGAGACAGCTACTCG
GTCTCACTCATCTGATGATC	CAGGAATTGGTCAAAACCGC	GACTTGAAGATGTCTTTGCA	AAGAGGTAGCACTCTTGGTC
GCGAACAACGGAGAAGACGC	TTGTTGGTGACACACCAAGA	GAGGTCGAAACGTACGTTCT	CGTGATCAGAAGTCCCTAAG
GAGATCAGGAAGATCTGGCG	AGGAAGTGCCCAGCATATAG		ATTCCAACACTGTGTCAAGC
GGATGAGGGAGTTGATTCTG	GTATTGGTTCATGGTCTCAA		
ATCTACCGAAGGAGAGACGG	GATGGAAAGGCATGGCTACA		

**A/Mallard PB2 mRNA**	**A/Mallard PB1 mRNA**	**A/Mallard PA mRNA**	**A/Mallard HA mRNA**
AGGTTTTGTAGACCTTTGGA	TGTTCCATGGCTGTATGGAG	GTTCGTTTCGATTTTCGGAT	TGAACTAGCTCGGTAGCATT
GTTTTAATCTTTCGACCTTT	GATGTGTTCTATTGACTGTG	TAAGTGTGTGCATATCGCGG	GCATATTTTCCCTGTTGAAG
AAGTGAACGGGACCGAAGGT	CACTTCCCTTTCTCTGAGTA	CCGAATACATGAAACAGACC	CTTCCATCAAGAATCCTGTG
CGGCGTATTTTAACCTGATT	TCCAGTCTCGGTGTTTGTTG	CCCCGTTCATCAATAAAGTG	GTAGGGCATCTATTAGTGTG
AGCACTGAGATCTGAATGGC	CAATTGGATTGAGTTGGGGT	TCGGTGTTTCAATAATGCAT	AGACATCGCAATGAGGATCC
TGATGACATCTTGTGCTTCT	CTCGTTATCCTCAGGCAATG	TCTCTCCCTTCAATTATTTC	CAAAGAGATCCCATGTCTCA
TCGTTTGGGAAAACGACCTC	AATCTGTTTGTGCATATCCG	GTTGCAGATACTATTCACCA	CTGAAAGCATTGCTTCGCTC
CTCTGATGTCAATATCCTGG	ACTTCCATTGTTTCAAGACA	AATTTAGGCTTATCGACTCC	GGTACATCGTAAGGATAGCA
CCACCATCAAAGGAGCAATC	TCAGTTTATCCACTCTTGTT	CGGTTCTCTTTGTAGTCATA	GGATCGAAGGGATGCATAGT
AGTTCTCTCTCCAACATGTA	TTCAATGTCCAGTCATAGGT	GTGTCACTCCAATTTCAATG	CAATGTGCCTGATGATGCAA
TGGTAGGAATCTGGTTTTGC	TCTATAGTGTTGGCCAAAGC	TAGTATATGTGAACTTCCCT	CCAAGTGAAACCTTCAGTGA
CTCGATATACACGCTGCTTG	GTTAGACCGTTCGATCTGAA	TTTCCTCTCCAGTGAATGAA	TGAAGAAACCGCTAGCAGGT
TCCCTTGAGTCAAATGCAAC	TATTAGTCTCCCTGATTCAT	TTCATCAAGAGTGTAGTCCG	TCACGTTCAACACTGGGTAT
GTGTGTACATTTGTTCCCAG	CTTACTCTTCTCTTTCGTTG	TTTTGATTCTTGCCCTACTC	CCCAGATGTATAATTTGTCA
ACATCATCATTTCTCACCTC	CCATTTTCTTGGTCATGTTG	TTTCCTGCCTTATAGTGAAC	TTTGTGCTCGGGTGGTGAAC
ATTTCTGGCAGCAATAATCA	TTCTTCCCTATTGTTCTTTG	CTCTCGGACTGACGAAAGGA	TCCTGAGGCTTGAACATACA
TGATACTGTTGCTCTCCTAA	CATTGTGTTCAGTGTCAGTG	TTCTTCAATTGTCTCTTCGC	TCCTGGTAGAGACTGTGACT
TCTCCAAAAGCGAAGCCAAC	CATTCCGGGTGTTGCAATTG	CATGGTTCCTGTGATTTCAA	GGGATTATAGTCTGCTGGCT
CGCCAATTTGTGTACTATGG	AGTACACGAACCCTCTGATT	AAGTTCGGTGGGAGACTTTG	CCAGGGTCTAGATCCAATGT
AAGGATGTCCACCATCCTTA	CTCCTCGCTAATGTTTCGAC	TCTAAAGTTTTCAAGGCTGG	ATGCTTATTCTGCCAGACTG
TGCTCTTCTGTTGGGTTTTG	ACTGCTCAAGTTTCTCACAG	GTTCGAATCCATCCACATAG	CAGGTTTGACTATTGTCCAG
TGCTTTGCATATATCCACAG	TTTTTCTCATTCCCTCCAAC	GAGAAAGCTTGCCCTCAATG	CTATTGATCACCAGTACGTC
TGAACTGATTCTTAGACCCA	CACGACATTTGCCAGTTTAG	GCGTTCACTTCTTTTGACAT	AGGAGCGATTAGGTTTCCAT
AGTGAAACCTCCAAAGCTGA	GTGAGTTAGTCATCATCTTT	GTTGTCTTCAGAAATGGCTC	CAGTGCGCATCTTGAAGTAG
TTTCAATGTTTGGAGGTTGC	GTAAAGGAGAGCTCTGTGTC	CATCAGGTAGCTTGAGAGGG	GACCTCATTATCGAGCTTTT
TATTAGTTGGATCAGCCTTC	CTTGGTGTTGTCTCCAGTAA	GGAACTTCGACCGTTGAGAG	ACAGGTGTCAATAGGTGCAT
CGATTGACTGCTCGTCTCTC	ATCCGAGGATTCTGATTCTC	AATTTAAGGGCATCCATCAG	TTGGGGTGATGCATTCAGAA
AACCATTGCCACTATGATCG	TATATGTTATCATTGCCAGA	CATGACTCGGGTCTTCGATG	CTTGTCATTGGGGATGCTTC
TTATCATGCAATCCTCTTGT	CATTCAGGTTGGTTTCTTGT	TGATTGCATCATATAGCGGT	GTGATCTTGTTCACATTTTG
TTCAAATCACCTCGTACTGC	GCGATACTCAGGACATTTCT	CCAGCCGAAAAATGTCTTCA	CATATTTGGGACATGCTCCG
			AACTTCAGGGTGTTCTGCTT
			TACATTCCGCATTCCTGTTG
			AATAGGCCTCTGGTTTGCTT
			CTATAAAGCCTGCTATTGCA
			ATCTATCATTCCTTCCCATC
			GATGCCTGAAGCCATACCAA
			TGTCCTGTACCTTCGGAATT
**A/Mallard NP mRNA**	**A/Mallard NA mRNA**	**A/Mallard M mRNA**	AGTGCTTTTAAGGTCTGCTG
TTCTTCCAACAGATGCTCTG	GGGGTATTGCACTCATTTTG	CTGACGGGACGATAGAGAGA	TTGATCTGGTCAATGGCTGC
AACCTTCCAATTCCACCAAC	ATGGCACCACTTGATTGTTC	CCCTGCAAAGACATCTTCAA	AATCACTCTGTTCAATTTCC
TTCAGTGCACATCTGTATGT	TCTGACATTGCGGTTTTGAC	TGAGAGCCTCGAGATCGGTG	GGAACTTCTCATTCGTCTTT
CTTCATAGTCGCTGAGTTTG	GCTGCATGACACATAAGGTT	GGTCTTGTCTTTAGCCATTC	CTCGGAGAATTCCTTTTCGA
TTATGCTGTTCTGGATCAGC	TTTGTTATCCAGCGTGGTTC	CTTAGTCAGAGGTGACAGGA	TTCTCAAGGTTCTGAATCCT
TGGGATGTTCTTCCAGATAT	CTGGACCATGCTATGCACAC	AGCGTGAACACAAATCCTAA	GAGTTCCGCATTATAAGACC
TAGATTGGACCTCCAGTTTT	TAGCAGTCGCATTTCTATCA	ATGTTGTTTGGGTCTCCATT	TGTATGCTGATTCTCTAGGG
ATCAGATGAGTGAGACCAGC	TCTGATTCCTGAGTTCTGAG	CTTCCTGTACAGTTTGACTG	GTTCATTTCTGAATCAGTCA
ATCATTCAGGTTGGAATGCC	AGTTCCATTGATGCAAACGC	CATACAACTGGCAAGTGCAC	CCTTCTGGTCTTTTCAAACA
CTCTCGTTCTCTGGTATGTG	CTTTCCCCTCTCTAATGAAT	CCATCCTGTTGTATATGAGA	CATGTCTTCAGCATTTTCCC
CATTCTTGGGTCCATACCAG	CTGACAATGGGCTGACATGA	AAAAGCCACTTCTGTGGTCA	CTATGCAAGCATTGTCACAC
TTGATCCTTGCATCAGAGAG	CAATGCTATAGTCTGCCATA	ATGTTGTTTGGGTCTCCATT	
AATTAGTTCCATCACCATCG	GATAGTTCTTCCCATCCAAA	CTTCCTGTACAGTTTGACTG	**A/Mallard NS mRNA**
AAGTCCGTACACACAAGCAG	CATAACCTGAGCGTGAATCC	CATACAACTGGCAAGTGCAC	GCGAAGCCGGTCAAGGAATG
AAGTCATATCCACTGGCCAC	CCTCTTATCAACTCCACATA	CCATCCTGTTGTATATGAGA	CTCTTAGGGACTTCTGATCA
CAGAGAGTATCCTTCTCTCT	CATACTCTAGTCTCCTGTGG	AAAAGCCACTTCTGTGGTCA	CAGACCAAGAGTGCTACCTC
GACGAAAAGGATCTATTCCG	TAGGATGGCAACCTGCATGA	CTGTGAATCAGCAATCTGCT	CACGAGTAGCTGTCTCGATG
GAAGACCTGGCTGTTTTGAA	TCCTTTCTATTATGATTGGT	TCTAATTAGTGGGTTGGTGG	AGAATCCGCTCCACTATTTG
GGATTCTCATTTGGTCTAAT	TTCAAATACACTATCTCCGT	CTGACTAGCAACCTCCATGG	TAAGTGCCTCATCAGATTCT
TGATACTCTCAGGTCTTCAA	ACTTCTTTTTCTATGGTAGT	AATTGTTCTCATCGCCTGCA	GGCACTGAAGCAATAGTCAT
GATAGTTGTCCTCTTGGGAC	TTCCTGTATTCTACCATTTT	CACTGGAGCTAGGGTGAGTT	GTCAGTTAGGTAGCGTGAAG
GCTTCTCAATTCGAGAGTAC	TCCATCGGTCATTACTACTG	AGAAGATCATCTTTCAGACC	TTGACATTTCCTCAAGAGTC
TGGTTCTTATAGCCCAATAT	TATTTTAGTATCAGCCCTTC		GGCATGAGCATGAACCAGTC
CCCTTTCGAAGGGAAGATTT	TCAACAAGCATCCCATCATA		TTATCCATTATTGCCTGGTC
TGTAAATGCCGCCATAATGG	TATTTTGAGACCATGAACCA		GTTTGCTTTCAATATGATGT
CACTTTCCATCATCCTTATG	CCTGAGCACACATAACTAGA		GCCGGTCAAAAATCACACTG
AGGACTTATGGGCCACTGAT	CCACTAATGACCCTGAAAGT		GCTCTAAGTAGTATTAGGGT
TCAGTAGCATTCTGGCGTTC	GACGATACTATTTGAGGTCC		AATTGCTCCTTCTTCTGTGA
TTTCTCGTACTTGATCCATC	GGAACATTCCTCTATATGCT		GGTAACGGTGAGATTTCTCC
ATTTCAGCATTCCCAGGATT	ACGTCTGGATATCGAGGATA		CATCAGTATGTCCTGGAAGA

Supplementary Table 1: FISH probe sets. DNA oligonucleotide sequences for detection of vRNA and mRNA of A/Panama and A/Mallard.

Supplementary Table 2

MSC Rank 2

Experiment		Predicted	
Segments	n	Segments	n
**23**	23	**27**	38
**35**	17	**37**	16
**37**	17	**23**	12
**27**	15	**35**	10

MSC Rank 3

Experiment		Predicted	
Segments	n	Segments	n
**237**	14	**237**	31
**127**	7	**127**	12
**257**	5	267	7
234	5	**257**	4

MSC Rank 4

Experiment		Predicted	
Segments	n	Segments	n
**1237**	10	2378	16
1257	5	**2367**	8
1267	5	**1237**	7
**2367**	5	2357	5

MSC Rank 5

Experiment		Predicted	
Segments	n	Segments	n
**12367**	9	**12367**	10
23467	8	24567	7
23678	7	**12378**	5
**12378**	7	12468	5

MSC Rank 6

Experiment		Predicted	
Segments	n	Segments	n
**123678**	19	**123467**	21
**123467**	13	**123678**	16
123457	10	123578	10
**123567**	10	**123567**	9

MSC Rank 7

Experiment		Predicted	
Segments	n	Segments	n
**1234567**	41	**1234567**	57
**1235678**	41	**1235678**	31
**1245678**	26	**1245678**	25
**1234678**	17	**1234678**	19

MSC Rank 8

Experiment		Predicted	
Segments	n	Segments	n
**12345678**	355	**12345678**	319

Supplementary Table 2: Comparison between experimental and predicted abundances n of the four most abundant MSCs in each rank for Cell 18. The four most abundant experimental and predicted MSCs in each rank are listed. MSCs that occur at a given rank in both the experimental and predicted MSCs are bold. Especially for the higher ranks 5 to 7 there is a good agreement between experiment and model.
