# Supplementary material for: Selective flexible packaging pathways of the segmented genome of influenza A virus
Source: Nat Commun. 2020 Aug 28;11:4355. doi: 10.1038/s41467-020-18108-1 (PMC7455735; doi:10.1038/s41467-020-18108-1)
Supplement: Supplementary file 3 — Reporting Summary [file 41467_2020_18108_MOESM3_ESM.pdf]

nature research

Corresponding author(s): Thorsten Wolff, Andreas Herrmann

Last updated by author(s): Jul 24, 2020

Reporting Summary

Nature Research wishes to improve the reproducibility of the work that we publish. This form provides structure for consistency and transparency in reporting. For further information on Nature Research policies, see Authors & Referees and the Editorial Policy Checklist .

Statistics

For all statistical analyses, confirm that the following items are present in the figure legend, table legend, main text, or Methods section.

n/a	Confirmed	
☐	☒	The exact sample size (n) for each experimental group/condition, given as a discrete number and unit of measurement
☐	☒	A statement on whether measurements were taken from distinct samples or whether the same sample was measured repeatedly
☐	☒	The statistical test(s) used AND whether they are one- or two-sided<br>*Only common tests should be described solely by name; describe more complex techniques in the Methods section.*
☒	☐	A description of all covariates tested
☐	☒	A description of any assumptions or corrections, such as tests of normality and adjustment for multiple comparisons
☐	☒	A full description of the statistical parameters including central tendency (e.g. means) or other basic estimates (e.g. regression coefficient) AND variation (e.g. standard deviation) or associated estimates of uncertainty (e.g. confidence intervals)
☐	☒	For null hypothesis testing, the test statistic (e.g. F, t, r) with confidence intervals, effect sizes, degrees of freedom and P value noted<br>*Give P values as exact values whenever suitable.*
☒	☐	For Bayesian analysis, information on the choice of priors and Markov chain Monte Carlo settings
☒	☐	For hierarchical and complex designs, identification of the appropriate level for tests and full reporting of outcomes
☒	☐	Estimates of effect sizes (e.g. Cohen's d, Pearson's r), indicating how they were calculated

Our web collection on statistics for biologists contains articles on many of the points above.

Software and code

Policy information about availability of computer code

Data collection	VisiView Software 3.x<br>ImageJ
Data analysis	Stellaris probe designer 4.0,<br>NUPACK: J. N. Zadeh, C. D. Steenberg, J. S. Bois, B. R. Wolfe, M. B. Pierce, A. R. Khan, R. M. Dirks, N. A. Pierce. NUPACK: analysis and design of nucleic acid systems. J Comput Chem, 32:170–173, 2011.<br>igraph<br>Graphpad Prism v6.0d<br>ImageJ 1.52b<br>FISH-quant (compiled version: 2017-11-07; 12:57h)<br><br>R 3.2.4<br>R Studio 1.1.463<br>igraph 1.0.0<br>Custom R script: MegaFISH17b.R

For manuscripts utilizing custom algorithms or software that are central to the research but not yet described in published literature, software must be made available to editors/reviewers. We strongly encourage code deposition in a community repository (e.g. GitHub). See the Nature Research guidelines for submitting code & software for further information.

Data

Policy information about availability of data

All manuscripts must include a data availability statement. This statement should provide the following information, where applicable:

- Accession codes, unique identifiers, or web links for publicly available datasets
- A list of figures that have associated raw data
- A description of any restrictions on data availability

The authors declare that the data supproting the findings of this study are available within the paper and its supplementary information files.

Field-specific reporting

Please select the one below that is the best fit for your research. If you are not sure, read the appropriate sections before making your selection.

[x] Life sciences   [ ] Behavioural & social sciences   [ ] Ecological, evolutionary & environmental sciences

For a reference copy of the document with all sections, see nature.com/documents/nr-reporting-summary-flat.pdf

Life sciences study design

All studies must disclose on these points even when the disclosure is negative.

Sample size	Sample sizes were calculated and evaluated on the basis of the scattering dimensions in order to generate statistically significant, publication quality values. For each experiment ample sizes are indicated in the legends (Figs. 2-5, Extended Data Figs. 6, 7, 11).
Data exclusions	No data were excluded from analysis.
Replication	All attempts to reproduce findings were successful. Data in Figs. 1, 2,5 and 6,and Supp. Figs 1, 8, 9, 10 and 13 were acquired by three or four independent experiments as indicated in legends. To reduce potential false-positive signals each vRNA was targeted twice along the cycles, once with Atto550 and once with STAR635P labelled probe sets (see Results). 207 cells were identified for Fig. 1 and Supp. Fig. 1, 218 cells for Supplemental Figure 10. Colocalization of vmRNA and vRNA in Supp. Fig. 11 was analyzed for one representative position, but distribution patterns of RNAs were similar for all three experiments. Supp. Figs. 2, 3, 4 and 5 were performed once for visualization of experimental setups and of their bottlenecks, and to demonstrate probe specificity.
Randomization	The staining order of the different vRNA/vmRNA signals was randomly varied in the different replicates to correct for any potential bias caused by an “early” or “late” staining during the MuSeq-FISH cycles. For image analyses, 69 cells out of 207 cells were used for A/Panama, and 54 cells out of 218 cells were chosen for A/Mallard. The remaining cells were either excluded because of not being infected, or cells were incomplete due to crossing of image borders. Randomization was not performed since all fully visible and infected cells were used for image analysis. As a negative control of the algorithm, colocalization of spots of two superimposed copies of the same image was analysed, whereby these two copies were rotated by 90° against each other. The resulting colocalization was low. The highest rank of MSCs observed was 2, with less than 1% of the spots of one image colcalized with spots of the other image rotated by 90°.
Blinding	Scientists were not blinded to specimen allocation during data collection and/or analysis.<br>The experimental procedure of MuSeq-FISH , the central method of the work, is complex and requires numerous immediate following-on and very time-consuming protocol steps. The key experiments to obtain the raw data (i.e. excluding image analysis etc.) require about 90 hours per independent experiment. For this reason, each experiment was performed by 2 persons together, who took turns in performing the experiment. The repetition of such an experiment by an independent group of 2 persons would have exceeded the manpower of the laboratory. Experiments in Supplementary Figs. 8, 9 and 10 were conducted by different individuals at different times.

Reporting for specific materials, systems and methods

We require information from authors about some types of materials, experimental systems and methods used in many studies. Here, indicate whether each material, system or method listed is relevant to your study. If you are not sure if a list item applies to your research, read the appropriate section before selecting a response.

Materials & experimental systems

n/a	Involved in the study	
[ ]	[x]	Antibodies
[ ]	[x]	Eukaryotic cell lines
[x]	[ ]	Palaeontology
[x]	[ ]	Animals and other organisms
[x]	[ ]	Human research participants
[x]	[ ]	Clinical data

Methods

n/a	Involved in the study	
[x]	[ ]	ChIP-seq
[x]	[ ]	Flow cytometry
[x]	[ ]	MRI-based neuroimaging

Antibodies

Antibodies used	FITC-conjugated anti-NP antibody (MAB8257F, Merck Millipore, Darmstadt, Germany)
Validation	Species and specificity, as well as the use for specific applications were confirmed/investigated by the manufacturer (Merck Millipore, Darmstadt, Germany).

Eukaryotic cell lines

Policy information about cell lines

Cell line source(s)	A549, ATCC CCL-185; MDCK type II, ECACC 00062107
Authentication	Cell lines were not separately authenticated.
Mycoplasma contamination	All cell lines used within this study have been tested negative for mycoplasma contaminations with PCR.
Commonly misidentified lines (See ICLAC register)	No commonly misidentified cell lines were used.
