# Supplementary material for: Selective flexible packaging pathways of the segmented genome of influenza A virus
Source: Nat Commun. 2020 Aug 28;11:4355. doi: 10.1038/s41467-020-18108-1 (PMC7455735; doi:10.1038/s41467-020-18108-1)
Supplement: Supplementary file 4 — Source Data [file 41467_2020_18108_MOESM4_ESM.zip › 231972_2_supp_4801007_q773ck.pdf]

```
### ----------------------------------------------------------
### MEGA-FISH colocalization script using FISH-QUANT output
### ----------------------------------------------------------

# Can be used to colocalize up to 8 different targets, (MUST BE
modified for higher numbers of targets)!!

# GENERAL SETTINGS (not loop-specific)
virustype <- "influenza" # can be influenza or hanta, this modifies
some specific settings
mainfolder <- "C:/Users/sprisner/Desktop/ColocTest/" # Output for
hanta specific testing
readcells <- T # expects a batch-file with cell segmentation
cellthresh <- 10 # analysis: threshold for displaying only cells
with more spots (all channels) than threshold
#targetnames <- c("Gc","N") # Hanta

targetnames <- c("PB2","PB1","PA","HA","NP","NA","M","NS") # specify
the names of your channels in order of identifiers

stackNumbRegEx <- "_s.*_" #RegEx pattern to identify stack number
i.e "_s10_cor"
# END GENERAL SETTINGS

### BEGIN VARIABLE SETTINGS

# directory - VERY IMPORTANT: trailing slash after folder
folderlist <- list("//TS412-MOLBP/Shared/Simon/FQ_160404/
GoodCellsCombinations/AuswertungSegInMscs/")

expnr1 <- 1 # experiment number
picnr1 <- 2 # picture number

# Box /circle size for colocalization
usecircle <- T # if true, will use a circle (cylinder) instead of
box for colocalization
xt <- 300
yt <- 300
zt <- 1000
rt <- c(300)

# Picture settings
xyres <- 130 # how many nm per px in xy?
zres <- 300 # same in z-direction
xypx <- 1024 # number of pixels in xy
zsl <- 26 # number of z-slices

# Negative control
mock <- F

# Use nucleus? - True keeps nuclear spots!
nucleus <- F

# Take equal numbers of points for each segment (based on weakest
```

```
segment)? T = yes
# caution: works on all images, might disadvantage entire individual
images
equalnumbers <- F

# Scramble dataframe before detection (recommended)
scrambledf <- T

# Perform a second colocalization run using the newly calculated
centroid
secondcoloc <- F
secondcoloc_threshold <- 2 # minimal rank of MSC to be re-
colocalized

# Use a second colour to double-check
use_replicates <- T # if true, will look for segment replicates

### END SETTINGS
options(stringsAsFactors = FALSE)

# Populate settings list for looping
new <- list(expnr1 = expnr1, picnr1 = picnr1,
                    usecircle = usecircle, xt = xt, yt = yt, zt =
zt, rt = rt, xyres = xyres, zres = zres,
                    xypx = xypx, zsl = zsl, mock = mock, nucleus =
nucleus, equalnumbers = equalnumbers,
                    scrambledf = scrambledf,
                    secondcoloc = secondcoloc, secondcoloc_threshold
= secondcoloc_threshold,
                    use_replicates = use_replicates)
maxlen <- 1
for (i in new) {
  if (length(i) > maxlen) maxlen <- length(i)
}
settingsets <- list()
for (i in 1:maxlen) {
  settingsets[[i]] <- list()
  if (is.na(expnr1[i])) settingsets[[i]]$expnr1 <- tail(expnr1,n=1)
else settingsets[[i]]$expnr1 <- expnr1[i]
  if (is.na(picnr1[i])) settingsets[[i]]$picnr1 <- tail(picnr1,n=1)
else settingsets[[i]]$picnr1 <- picnr1[i]
  if (is.na(usecircle[i])) settingsets[[i]]$usecircle <-
tail(usecircle,n=1)
    else settingsets[[i]]$usecircle <- usecircle[i]
  if (is.na(xt[i])) settingsets[[i]]$xt <- tail(xt,n=1) else
settingsets[[i]]$xt <- xt[i]
  if (is.na(yt[i])) settingsets[[i]]$yt <- tail(yt,n=1) else
settingsets[[i]]$yt <- yt[i]
  if (is.na(zt[i])) settingsets[[i]]$zt <- tail(zt,n=1) else
settingsets[[i]]$zt <- zt[i]
  if (is.na(rt[i])) settingsets[[i]]$rt <- tail(rt,n=1) else
settingsets[[i]]$rt <- rt[i]
  if (is.na(xyres[i])) settingsets[[i]]$xyres <- tail(xyres,n=1)
else settingsets[[i]]$xyres <- xyres[i]
```

```
  if (is.na(zres[i])) settingsets[[i]]$zres <- tail(zres,n=1) else
settingsets[[i]]$zres <- zres[i]
  if (is.na(xypx[i])) settingsets[[i]]$xypx <- tail(xypx,n=1) else
settingsets[[i]]$xypx <- xypx[i]
  if (is.na(zsl[i])) settingsets[[i]]$zsl <- tail(zsl,n=1) else
settingsets[[i]]$zsl <- zsl[i]
  if (is.na(mock[i])) settingsets[[i]]$mock <- tail(mock,n=1) else
settingsets[[i]]$mock <- mock[i]
  if (is.na(nucleus[i])) settingsets[[i]]$nucleus <-
tail(nucleus,n=1) else settingsets[[i]]$nucleus <- nucleus[i]
  if (is.na(equalnumbers[i])) settingsets[[i]]$equalnumbers <-
tail(equalnumbers,n=1)
    else settingsets[[i]]$equalnumbers <- equalnumbers[i]
  if (is.na(scrambledf[i])) settingsets[[i]]$scrambledf <-
tail(scrambledf,n=1)
    else settingsets[[i]]$scrambledf <- scrambledf[i]
  if (is.na(secondcoloc[i])) settingsets[[i]]$secondcoloc <-
tail(secondcoloc,n=1)
    else settingsets[[i]]$secondcoloc <- secondcoloc[i]
  if (is.na(secondcoloc_threshold[i])) settingsets[[i]]
$secondcoloc_threshold <- tail(secondcoloc_threshold,n=1)
    else settingsets[[i]]$secondcoloc_threshold <-
secondcoloc_threshold[i]
  if (is.na(use_replicates[i])) settingsets[[i]]$use_replicates <-
tail(use_replicates,n=1)
    else settingsets[[i]]$use_replicates <- use_replicates[i]
}

# load libraries
library(ggplot2)
library(gstat)
library(gplots)
library(dplyr)
library(tidyr)
library(gridExtra)
library(xlsx) # for excel output
library(proto) #necessary for strapply
library(gsubfn) #necessary for strapply
library(car)
#library(svglite)
library(stringr)
library(reshape2)

# run until here for summary analysis

# main for-loop to cycle through all setting sets
fcount <- 0
for (f in folderlist) {
    # folder output
    cat("\n----------------------- NEW DIR
-------------------------------","\n")
    setwd(f) # Folder for spot detection files
    cat("Working dir: ",getwd(),"\n") # user output
    fcount <- fcount + 1
```

```
    scount <- 0
    for (s in settingsets) {
        # s is current settingset
        save <-
c("folderlist","s","f","settingsets","scount","fcount",

"virustype","mainfolder","readcells","targetnames","cellthresh") #
save these variables from deletion
        rm(list = ls()[!(ls()%in%save)]) # clear up memory for next
run

        # get identifier for each setting set to save in different
folders
        scount <- scount + 1
        sdir <- paste(f,"NewResults_",scount,sep="")

        # settings output
        cat("-------------------- NEW SETTINGS
------------------------------","\n")

        ### LEGACY ###
        ysize <- s$xypx

        # initialize alignment vectors
        x <- rep(0,16)
        y <- rep(0,16)
        z <- rep(0,16)

        # Read files
        if(file.exists("alignment.txt") == T) {
          alignment_file <- read.table("alignment.txt", header =
FALSE, fill = TRUE)

            x[1] <- as.numeric(as.character(alignment_file[2,1]))
# PB2
            y[1] <- as.numeric(as.character(alignment_file[3,1]))
            z[1] <- as.numeric(as.character(alignment_file[4,1]))
            x[2] <- as.numeric(as.character(alignment_file[6,1]))
# PB1
            y[2] <- as.numeric(as.character(alignment_file[7,1]))
            z[2] <- as.numeric(as.character(alignment_file[8,1]))
            x[3] <- as.numeric(as.character(alignment_file[10,1]))
# PA
            y[3] <- as.numeric(as.character(alignment_file[11,1]))
            z[3] <- as.numeric(as.character(alignment_file[12,1]))
            x[4] <- as.numeric(as.character(alignment_file[14,1]))
# HA
            y[4] <- as.numeric(as.character(alignment_file[15,1]))
            z[4] <- as.numeric(as.character(alignment_file[16,1]))
            x[5] <- as.numeric(as.character(alignment_file[18,1]))
# NP
            y[5] <- as.numeric(as.character(alignment_file[19,1]))
            z[5] <- as.numeric(as.character(alignment_file[20,1]))
```

```
        x[6] <- as.numeric(as.character(alignment_file[22,1]))
# NA
        y[6] <- as.numeric(as.character(alignment_file[23,1]))
        z[6] <- as.numeric(as.character(alignment_file[24,1]))
        x[7] <- as.numeric(as.character(alignment_file[26,1]))
# M
        y[7] <- as.numeric(as.character(alignment_file[27,1]))
        z[7] <- as.numeric(as.character(alignment_file[28,1]))
        x[8] <- as.numeric(as.character(alignment_file[30,1]))
# NS
        y[8] <- as.numeric(as.character(alignment_file[31,1]))
        z[8] <- as.numeric(as.character(alignment_file[32,1]))
        # 2nd color
        x[9] <- as.numeric(as.character(alignment_file[34,1]))
# PB2
        y[9] <- as.numeric(as.character(alignment_file[35,1]))
        z[9] <- as.numeric(as.character(alignment_file[36,1]))
        x[10] <- as.numeric(as.character(alignment_file[38,1]))
# PB1
        y[10] <- as.numeric(as.character(alignment_file[39,1]))
        z[10] <- as.numeric(as.character(alignment_file[40,1]))
        x[11] <- as.numeric(as.character(alignment_file[42,1]))
# PA
        y[11] <- as.numeric(as.character(alignment_file[43,1]))
        z[11] <- as.numeric(as.character(alignment_file[44,1]))
        x[12] <- as.numeric(as.character(alignment_file[46,1]))
# HA
        y[12] <- as.numeric(as.character(alignment_file[47,1]))
        z[12] <- as.numeric(as.character(alignment_file[48,1]))
        x[13] <- as.numeric(as.character(alignment_file[50,1]))
# NP
        y[13] <- as.numeric(as.character(alignment_file[51,1]))
        z[13] <- as.numeric(as.character(alignment_file[52,1]))
        x[14] <- as.numeric(as.character(alignment_file[54,1]))
# NA
        y[14] <- as.numeric(as.character(alignment_file[55,1]))
        z[14] <- as.numeric(as.character(alignment_file[56,1]))
        x[15] <- as.numeric(as.character(alignment_file[58,1]))
# M
        y[15] <- as.numeric(as.character(alignment_file[59,1]))
        z[15] <- as.numeric(as.character(alignment_file[60,1]))
        x[16] <- as.numeric(as.character(alignment_file[62,1]))
# NS
        y[16] <- as.numeric(as.character(alignment_file[63,1]))
        z[16] <- as.numeric(as.character(alignment_file[64,1]))
    }

    # Read files
    df <- list()
    count <- 1
    spotcount <- c()
    segments <- c(0)

    # if summary files exist, split them up to generate standard
```

```
files
        if (readcells == T) {
          for (i in list.files(pattern="all_spots")){
            # read in summary file
            sumf <- read.table(i, dec=".", header = FALSE, sep =
"\t", as.is = T,
                               col.names = paste0("V",seq_len(46)),
fill = TRUE)

            header <- sumf[1:14,]
            fillrow <- header[14,]
            fillrow[1:29] <- fillrow[3:31]
            for (r in 1:5)
              header <- rbind(header, fillrow)

            data <- sumf[15:nrow(sumf),]

            data[,32] <- data[,1] # save differing filenames
            data[,46] <- data[,35] # save nucleus information
            data[,39] <- as.integer(sapply(data[,2], substr, 6, 9))
# save cells, removing "Cell_"

            data[,1:31] <- data[,4:34] # reorder changed columns

            nfiles <- unique(data[,32])

            for (j in 1:length(nfiles)) {
              newfile <- rbind(header, data[data[,32] ==
nfiles[j],])
              newfile[5,1] <- "IMG_Raw"
              newfile[5,2] <- nfiles[j]

              filenr <- substr(nfiles[j], 1, 2)

              write.table(newfile, file=paste(filenr,
"_segment_spots.txt", sep=""),
                          col.names=F, row.names=F, sep = "\t",
quote = F)
            }

            dir.create("summaryfiles")
            file.rename(from=i,
                        to=paste("summaryfiles/",i,sep=""))

#file.rename(i,paste(nfiles[1],"_summaryfileused.txt",sep=""))
          }
        }

        for (i in list.files(pattern="spots")){
          df1 <- NULL
          df_spots <- NULL

          cat("Reading in: ",i,"\n") #show user which files are
actually processed
```

```
          df1 <- read.table(i, dec=".", header = FALSE, sep = "\t",
as.is = T,
                            col.names = paste0("V",seq_len(46)),
fill = TRUE)

          # fill/initialize new columns and their names
          if (readcells == F) df1[,32] <- 0 # Filename
          df1[,33] <- count # Segment number
          df1[,34] <- F # used / unused by spot detection
          df1[,35] <- 0 # MSC identifier (starting from 1)
          df1[,36] <- 0 # colocalization rank / number of segments
in one msc
          df1[,37] <- s$expnr1 # experiment number
          df1[,38] <- s$picnr1 # picture number
          #df1[,38] <- fcount # picture number
          if (readcells == F) df1[,39] <- 0 # cell number
          df1[,40] <- 0 # segment repetition
          df1[,41] <- 0 # MSC centroid x in nm
          df1[,42] <- 0 # MSC centroid y in nm
          df1[,43] <- 0 # x-distance to msc centroid in nm
          df1[,44] <- 0 # y-distance to msc centroid in nm
          df1[,45] <- F # double colour present
          if (readcells == F) df1[,46] <- F # in nucleus

          ### ACTUALLY READ THE DATA
          cols <- df1[19,] # correct lines for new version
          df_spots <- df1[20:nrow(df1),] # only use actual data
          df_spots[,32] <- df1[5,2] # save filename

          names(df_spots) <- cols # save remaining column names

          names(df_spots)[32] <- "filename"
          names(df_spots)[33] <- "segment"
          names(df_spots)[34] <- "used"
          names(df_spots)[35] <- "mscnr"
          names(df_spots)[36] <- "coloc_rank"
          names(df_spots)[37] <- "expnr"
          names(df_spots)[38] <- "picnr"
          names(df_spots)[39] <- "cellnr"
          names(df_spots)[40] <- "segrep"
          names(df_spots)[41] <- "centroidX"
          names(df_spots)[42] <- "centroidY"
          names(df_spots)[43] <- "distcentroidX"
          names(df_spots)[44] <- "distcentroidY"
          names(df_spots)[45] <- "doublecol"
          names(df_spots)[46] <- "in_nuc"

          # read segment from filename (reads only first character)
          segment <- as.numeric(substr(i,1,2))
          df_spots$segment <- segment
          # check if current segment is the first occurence or a
repetition of it
          a <- table(segments)
```

```
          if (length(a[names(a)==segment])==0) {
            df_spots$segrep <- 1
          } else {
            df_spots$segrep <- a[names(a)==segment] + 1
          }
          segments <- append(segments,segment)

          # drop x/y/z levels
          df_spots[,1] <- as.double(df_spots[,1])
          df_spots[,2] <- as.double(df_spots[,2])
          df_spots[,3] <- as.double(df_spots[,3])

          # add finished dataframe to dataframe-list
          df[[i]] <- na.omit(df_spots)
          count <- count + 1
        }
        filecount <- count - 1

        #segcount <- 8
        # Adjust target name vector
        #targetnames <- targetnames[1:segcount]

        # Adjust coordinates = "registration" ==> should be done
automatically in the future
        for (i in 1:filecount) {
          df[[i]][,2] <- df[[i]][,2] + x[i] * s$xyres
          df[[i]][,1] <- df[[i]][,1] + y[i] * s$xyres
          df[[i]][,3] <- df[[i]][,3] + z[i] * s$zres # + or minus
(reversed from MetaMorph)
        }

        # Rotate images in case of mock-control
        # 2: 90d, 3: 180d, 4: 270d, 5: flipx, 6: flipy, 7:
90d+flipx, 8: 90d+flipy above: 90d
        if (s$mock == T) {
          rotcenter <- s$xyres * s$xypx / 2
          for (i in 1:filecount) {
            # Rotate 90d
            if(i == 2 || i == 7 || i == 8 || i > 8) {
              print(paste("Segment:",i," - Rotation 90d"))
              df[[i]][,2] <- df[[i]][,2] + rotcenter
              df[[i]][,1] <- df[[i]][,1] + rotcenter

              oldx <- df[[i]][,2]
              oldy <- df[[i]][,1]

              df[[i]][,2] <- oldy  # x' = y
              df[[i]][,1] <- -oldx # y' = -x

              df[[i]][,2] <- df[[i]][,2] - rotcenter
              df[[i]][,1] <- df[[i]][,1] + rotcenter + 2*rotcenter
            }
            # Rotate 180d
            if(i == 3) {
```

```
      print(paste("Segment:",i," - Rotation 180d"))
      df[[i]][,2] <- df[[i]][,2] + rotcenter
      df[[i]][,1] <- df[[i]][,1] + rotcenter

      df[[i]][,2] <- -df[[i]][,2] # x' = -x
      df[[i]][,1] <- -df[[i]][,1] # y' = -y

      df[[i]][,2] <- df[[i]][,2] + rotcenter + 2*rotcenter
      df[[i]][,1] <- df[[i]][,1] + rotcenter + 2*rotcenter
    }
    # Rotate 270d
    if(i == 4) {
      print(paste("Segment:",i," - Rotation 270d"))
      df[[i]][,2] <- df[[i]][,2] + rotcenter
      df[[i]][,1] <- df[[i]][,1] + rotcenter

      oldx <- df[[i]][,2]
      oldy <- df[[i]][,1]

      df[[i]][,2] <- -oldy  # x' = -y
      df[[i]][,1] <- oldx # y' = x

      df[[i]][,2] <- df[[i]][,2] + rotcenter + 2*rotcenter
      df[[i]][,1] <- df[[i]][,1] - rotcenter
    }
    # Flip x
    if(i == 5 || i == 7) {
      print(paste("Segment:",i," - Flip x"))
      df[[i]][,2] <- -df[[i]][,2] + rotcenter*2  # x' = -x
    }
    # Flip y
    if(i == 6 || i == 8) {
      print(paste("Segment:",i," - Flip y"))
      df[[i]][,1] <- -df[[i]][,1] + rotcenter*2  # y' = -y
    }
  }
}

# Create common dataframe (allspots)
allspots <- na.omit(df[[1]])
for (i in 2:(filecount)) {
  allspots <- na.omit(rbind(allspots,df[[i]]))
}
cat("Dim(allspots): ",dim(allspots),"\n")

# Scramble dataframe before detection
if (s$scrambledf == T)
  allspots <- allspots[sample(nrow(allspots)),]

# Remove points in nucleus if option set
if (s$nucleus == F)
  allspots <- allspots[allspots$in_nuc == 0,]

# Remove points out of bounds
```

```
        max_x <- max(abs(x)) * s$xyres
        max_y <- max(abs(y)) * s$xyres
        max_z <- max(abs(z)) * s$zres
        allspots <- subset(allspots, (Pos_X > max_x) & (Pos_X <
(s$xypx*s$xyres - max_x))) # remove by x-value
        allspots <- subset(allspots, (Pos_Y > max_y) & (Pos_Y <
(s$xypx*s$xyres - max_y))) # remove by y-value
        #allspots <- subset(allspots, (Pos_Z > max_z) & (Pos_Z <
(s$zsl*s$zres - max_z))) # currently not used

        ### Find colocalization
        if (dim(allspots)[1]>0) { # skips detection and most output
if no spots found

        # Back up spots before 2nd color removal
        allspots_backup <- allspots

        # Check for second colour if option used, if not delete
second colour
        if (s$use_replicates == T) {
          doublesegstats <- matrix(,8,3) # statistics
          for (i in 1:nrow(allspots)) {
            if (allspots$segment[i] < 9) { # only for first set of
colours
              seg <- allspots$segment[i]
              doubleseg <- seg + 10
              subspots <- allspots[allspots$segment == doubleseg,]

              doublesegstats[seg,1] <-
nrow(allspots[allspots$segment == seg,])
              doublesegstats[seg,2] <- nrow(subspots)

              colocalising_spots <- (sqrt((subspots[,1] -
allspots[i,1])^2 +
                                         (subspots[,2] -
allspots[i,2])^2) < s$rt &
                                      abs(subspots[,3] -
allspots[i,3]) < s$zt)
              if (any(colocalising_spots) == T)
allspots$doublecol[i] <- T
              if (i%%100 == 0) print(paste("Remove double
spots:",i,"/",nrow(allspots)))
            }
          }
          allspots <- allspots[allspots$doublecol == T,] # only keep
spots in both colours
          allspots <- allspots[allspots$segment < 9,] # only keep
first colour

          # Statistics
          for (segm in 1:max(allspots$segment)) {
            doublesegstats[segm,3] <- nrow(allspots[allspots$segment
== segm,])
          }
```

```
        } else {
          allspots <- allspots[allspots$segment < 9,] # only keep
first colour
        }
        filecount <- length(unique(allspots$segment)) # update
filecount

        # only take best points for each segment if equal point
numbers - NEW
        if (s$equalnumbers == T) {
          # only take number of spots determined by smallest df ---
keep best spots
          spotmin <- min(table(allspots$segment))
          allspots <- allspots %>% group_by(segment) %>%
                        arrange(desc(SC_det)) %>%
filter(row_number(segment) <= spotmin)
          allspots <- as.data.frame(allspots) # ungroup
        }

        ## MAIN COLOCALIZATION START
        mscnr <- 1
        for (i in 1:nrow(allspots)) {
          if (allspots[i,"used"] == F) { # spot not used yet
            if (s$usecircle == T) {
              colocalising_spots <- (allspots[,"used"] == F &
                                       sqrt((allspots[,1] -
allspots[i,1])^2 +
                                              (allspots[,2] -
allspots[i,2])^2) < s$rt &
                                       abs(allspots[,3] -
allspots[i,3]) < s$zt)
            } else {
              colocalising_spots <- (allspots[,"used"] == F &
                                       abs(allspots[,1] -
allspots[i,1]) < s$yt &
                                       abs(allspots[,2] -
allspots[i,2]) < s$xt &
                                       abs(allspots[,3] -
allspots[i,3]) < s$zt)
            }
            allspots[colocalising_spots,"used"] <- T # mark as used
            allspots[colocalising_spots,"mscnr"] <- mscnr
            mscnr <- mscnr + 1
          }
          if (i%%100 == 0)

print(paste("Spot:",i,"/",nrow(allspots),"MSCnr:",mscnr))
        }
        ## MAIN COLOCALIZATION END

        # Calculate centroids and distances to it
        allspots <- allspots %>% group_by(mscnr) %>%
mutate(centroidX = mean(Pos_X),
```

```
centroidY=mean(Pos_Y),

distcentroidX=Pos_X - centroidX,

distcentroidY= Pos_Y - centroidY)

        # Inititally calculate colocalization rank
        allspots <- allspots %>% group_by(mscnr) %>%
mutate(coloc_rank = n())

        # Ungroup
        allspots <- as.data.frame(allspots)

        # Do a second colocalization round using the newly
calculated centroids
        if (s$secondcoloc == T) {
          # Momomers are allowed to be added to existing MSCs
          allspots[allspots$coloc_rank == 1,"used"] <- F
          for (i in 1:nrow(allspots)) {
            if (allspots[i,"coloc_rank"] >= s$secondcoloc_threshold)
{
              if (s$usecircle == T) {
                colocalising_spots <- (allspots[,"used"] == F &

sqrt((as.double(allspots[,1]) -
as.double(allspots[i,"centroidY"]))^2 +

(as.double(allspots[,2]) - as.double(allspots[i,"centroidX"]))^2)
                                                < s$rt &
                                         abs(allspots[,3] -
allspots[i,3]) < s$zt)
              } else {
                colocalising_spots <- (allspots[,"used"] == F &
                                         abs(allspots[,1] -
allspots[i,"centroidY"]) < s$yt &
                                         abs(allspots[,2] -
allspots[i,"centroidX"]) < s$xt &
                                         abs(allspots[,3] -
allspots[i,3]) < s$zt)
              }
              allspots[colocalising_spots,"used"] <- T # mark as
used
              allspots[colocalising_spots,"mscnr"] <-
allspots[i,"mscnr"]
            }
            if (i%%100 == 0)
              print(paste("2nd colocalization ---
Spot:",i,"/",nrow(allspots)))
          }
          # Calculate centroids and distances to it - 2nd time
          allspots <- allspots %>% group_by(mscnr) %>%
mutate(centroidX = mean(Pos_X),

centroidY=mean(Pos_Y),
```

```
distcentroidX=Pos_X - centroidX,

distcentroidY= Pos_Y - centroidY)
          allspots <- as.data.frame(allspots)

        }
        ### Colocalization and centroids END

        # Remove doubles - only MSCs will be kept where no segment
is present two or more times
        msc <- allspots[!
duplicated(allspots[,c("segment","mscnr")]),]

        # Fix colocalization rank (because of removed doubles)
        msc <- msc %>% group_by(mscnr) %>% mutate(coloc_rank = n())

        # Make dir for results (one for each settingset) and change
to it
        dir.create(sdir)
        setwd(sdir)
        settingsfile <- paste("Settings_",scount,".xlsx",sep="")
        if (file.exists(settingsfile))
          file.remove(settingsfile)
        write.xlsx2(s,settingsfile,sheetName = "Settings", append =
TRUE)

        # Save complete dataframe "msc" for later use (can be loaded
directly at this point)
        save(msc, file = "msc_postcoloc.Rda")

        ### Uncomment if summary analysis
         f <- folderlist[[1]]
         s <- settingsets[[1]]
         setwd(f)
         load("allmsc_r300_equalnof_goodcells_rept.Rda")
         filecount <- 8
         msc <- newmscs
         allspots <- newmscs

        ### ANALYSIS START ###

        # Histogram of found spots
        msctable <- table(msc[,"mscnr"])
        par(mfrow=c(1,1))
        histbreaks <- seq(0,filecount)
        histogram <- hist(msctable, breaks=histbreaks) # graphical
output
        amounts <- histogram$counts # vector of amounts of spots per
rank (1-8), e.g. monomers, dimers...

        histdf <- data.frame(breaks =
histogram$breaks[2:length(histogram$breaks)],
                             counts = histogram$counts, mids =
```

```
histogram$mids)
        p <- ggplot(histdf,aes(x = breaks, y = counts)) +
          geom_bar(stat="identity", fill = "black")  +
          geom_text(aes(y=counts, ymax=counts, label=counts),
                    position= position_dodge(width=0.9), vjust=2,
color="white") +
          theme_bw() +
          xlab('Complex size') +
          ylab('Counts') +
          labs(fill='Segment') +
          scale_y_continuous(expand = c(0,0)) +
          scale_x_continuous(breaks = histdf$breaks) +
          ggtitle("Sizes of detected multi-segment complexes")

ggsave(file=paste(s$expnr1,"_",s$picnr1,"_","hist_all",".png", sep =
""),
                 plot=p, width=10, height=8, dpi=160)

ggsave(file=paste(s$expnr1,"_",s$picnr1,"_","hist_all",".pdf", sep =
""),
               plot=p, width=10, height=8, dpi=160)

        # How does segment x behave in the spots? in % of total
spots of colocalization rank
        segment_in_mscs <- list()
        for (i in 1:filecount) {
          segment_in_mscs[[i]] <- list()
          for (j in 1:filecount) {
            segment_in_mscs[[i]][[j]] <- msc[msc$segment == i &
msc$coloc_rank == j,]
          }
        }

        # potential failure: division by amounts[0]! Test for by: if
(amounts[2]>0 & amounts[3]) { .. or similar
        plotdf <- data.frame(x = 0, y = 0, segment = 0, ref = 0)
        for (i in 1:filecount) {
          for (j in 1:filecount) {
            newline <- data.frame(x = j, y =
nrow(segment_in_mscs[[i]][[j]])/amounts[j], segment = i,
                                  ref = 0)
            newlineref <- data.frame(x = j, y = j/filecount, segment
= i, ref = 1)
            plotdf <- rbind(plotdf, newline)
            plotdf <- rbind(plotdf, newlineref)
          }
        }
        plotdf <- plotdf[2:nrow(plotdf),]

        segcount <- max(allspots$segment)
        levelnames <- c("1" = targetnames[1])
        for (l in 2:segcount) {
          levelnames <- c(levelnames, targetnames[l])
          names(levelnames)[l] <- l
```

```
        }

        p <- ggplot(plotdf,aes(x = x, y = y)) +
          geom_line(data = subset(plotdf, ref == 1),
                    linetype=2,color="grey",size=0.7)  +
          geom_line(data = subset(plotdf, ref == 0),
                    linetype=1,color="blue",size=1.2)  +
          geom_point(data = subset(plotdf, ref == 0)) +
          theme_bw() +
          xlab('Complex size') +
          ylab('% present') +
          scale_y_continuous(expand = c(0,0), limits = c(0,1.05)) +
          scale_x_continuous(expand = c(0,0), limits =
c(0.92,filecount+0.08)) +
          scale_color_manual(values=c("blue", "grey")) +
          ggtitle("Probabilites of segments being present in MSCs of
given rank") +
          guides(linetype=FALSE, color=FALSE)

        if (virustype == "hanta") {
            p <- p + facet_wrap(~segment, ncol = 3, labeller =
as_labeller(levelnames))
        } else {
            p <- p + facet_wrap(~segment, ncol = 4, labeller =
as_labeller(levelnames))
        }


ggsave(file=paste(s$expnr1,"_",s$picnr1,"_","segment_in_mscs",".png"
, sep = ""),
               plot=p, width=10, height=5, dpi=1600)

ggsave(file=paste(s$expnr1,"_",s$picnr1,"_","segment_in_mscs",".pdf"
, sep = ""),
               plot=p, width=10, height=5, dpi=1600)


        # Colocalization analysis
        matrices <- list()
        matrices_m <- list()
        matrices_abs <- list()

        # Count common spots in MSCs of all sizes (2-7) 1 is all, 8
is all except monomers
        for (i in 1:(filecount+1)) {
          matrices[[i]] <- matrix(,filecount,filecount)
          matrices_m[[i]] <- matrix(,filecount,filecount)
          matrices_abs[[i]] <- matrix(,filecount,filecount)
        }

        for (i in 1:filecount) { # all
          for (j in 1:filecount) {
            matrices_abs[[1]][i,j] <- dim(intersect(msc[msc$segment
== i,"mscnr"],msc[msc$segment == j,"mscnr"]))[1]
```

```
            matrices[[1]][i,j] <- matrices_abs[[1]][i,j] /
dim(msc[msc$segment == i,"mscnr"])[1]
            matrices[[1]][is.na(matrices[[1]])] <- 0
            matrices_m[[1]][i,j] <- max(matrices[[1]]
[i,j],matrices[[1]][j,i])
            matrices_abs[[1]][is.na(matrices_abs[[1]])] <- 0
          }
        }

        for (size in 2:filecount) { # specific MSC sizes (ranks)
          for (i in 1:filecount) {
            for (j in 1:filecount) {
              matrices_abs[[size]][i,j] <-
dim(intersect(msc[msc$segment == i & msc$coloc_rank ==
size,"mscnr"],

msc[msc$segment == j & msc$coloc_rank == size,"mscnr"]))[1]
              matrices[[size]][i,j] <- matrices_abs[[size]][i,j] /
dim(msc[msc[,"segment"] == i &

msc$coloc_rank == size,"mscnr"])[1]
              matrices[[size]][is.na(matrices[[size]])] <- 0
              matrices_m[[size]][i,j] <- max(matrices[[size]]
[i,j],matrices[[size]][j,i])
              matrices_abs[[size]][is.na(matrices[[size]])] <- 0
            }
          }
        }

        for (i in 1:filecount) { #  all MSCs except monomers
          for (j in 1:filecount) {
            matrices_abs[[filecount+1]][i,j] <-
dim(intersect(msc[msc$segment == i & msc$coloc_rank != 1,"mscnr"],

msc[msc$segment == j & msc$coloc_rank != 1,"mscnr"]))[1]
            matrices[[filecount+1]][i,j] <-
matrices_abs[[filecount+1]][i,j] / dim(msc[msc[,"segment"] == i &

msc$coloc_rank != 1,"mscnr"])[1]
            matrices[[filecount+1]][is.na(matrices[[filecount+1]])]
<- 0
            matrices_m[[filecount+1]][i,j] <-
max(matrices[[filecount+1]][i,j],

matrices[[filecount+1]][j,i])
            matrices_abs[[filecount+1]]
[is.na(matrices_abs[[filecount+1]])] <- 0
          }
        }

        # Produce heatmaps (first all, then except monomers, then
depending on segment size)
        png(file =
paste(s$expnr1,"_",s$picnr1,"_","heatmap_m_all",".png", sep = ""),
```

```
            width = 1200, height = 800)
        heatmap.2(matrices_m[[1]], dendrogram="none", trace="none",
symm = T,
                  col = bluered(200),
                  cellnote = round(matrices_m[[1]],2),
notecol="black",
                  density.info='none',
                  keysize=1, # Shrink key size
                  srtCol=0,     # Column label rotation
                  main='Colocalization for all MSCs (including
monomers)',
                  xlab='y in % of all MSCs containing x',
                  ylab='x in % of all MSCs containing y')
        dev.off()

        if (sum(matrices[[filecount+1]]) > 0) {
            png(file =
paste(s$expnr1,"_",s$picnr1,"_","heatmap_m_all_multimers",".png",
sep = ""),
                width = 1200, height = 800)
            heatmap.2(matrices_m[[filecount+1]], dendrogram="none",
trace="none", symm = T,
                      col = bluered(200),
                      cellnote = round(matrices_m[[filecount+1]],2),
notecol="black",
                      density.info='none',
                      keysize=1, # Shrink key size
                      srtCol=0,     # Column label rotation
                      main='Colocalization for all MSCs',
                      xlab='y in % of all MSCs containing x',
                      ylab='x in % of all MSCs containing y')
            dev.off()
        }

        if (filecount > 2) {
          for (size in 2:(filecount-1)) {
            if(sum(matrices[[size]]) > 0) {
                png(file =
paste(s$expnr1,"_",s$picnr1,"_","heatmap_m_",size,".png", sep = ""),
                    width = 1200, height = 800)
                heatmap.2(matrices_m[[size]], Rowv = NA, Colv = NA,
trace="none", symm = T,
                          col = bluered(200),
                          cellnote = round(matrices_m[[size]],2),
notecol="black",
                          density.info='none',
                          keysize=1, # Shrink key size
                          srtCol=0,     # Column label rotation
                          main=paste('Colocalization for MSCs of
size',size),
                          xlab='y in % of all MSCs containing x',
                          ylab='x in % of all MSCs containing y')
                dev.off()
            }
```

```
          }
        }

        # Investigate centroid measures
        mscr <- msc %>% distinct(mscnr, .keep_all = T) %>%
          select(mscnr,distcentroidX,distcentroidY,coloc_rank,
segment) %>%
          filter(coloc_rank > 1) %>%
          group_by(mscnr) %>%
          mutate(mscrad =
max(sqrt(distcentroidX^2+distcentroidY^2)))
        mscr$coloc_rank <- as.factor(mscr$coloc_rank)
        #mscr$segment <- as.factor(mscr$segment)
        mscr$segment <- factor(mscr$segment, levels=c(1:filecount),
labels=targetnames)

        p1 <- ggplot(data = mscr, aes(x =
sqrt(distcentroidX^2+distcentroidY^2), fill = segment)) +
                geom_density(alpha = 0.2) +
                theme_bw() +
                expand_limits(x = 0, y = 0) +
                guides(fill=FALSE) +
                labs(x="Distance to centroid of MSC [nm]",y="# of
spots",fill='Segment')

        p2 <- ggplot(data = mscr, aes(x =
atan2(distcentroidY,distcentroidX)*57.2958, fill = segment)) +
                geom_density(alpha = 0.2) +
                theme_bw() +
                expand_limits(x = 0, y = 0) +
                labs(x="Angle to centroid of MSC",y="# of
spots",fill='Segment')

        p <- grid.arrange(p1, p2, ncol=2)

ggsave(file=paste(s$expnr1,"_",s$picnr1,"_","centr_measures",".png",
sep = ""),
               plot=p, width=10, height=5, dpi=1600)

ggsave(file=paste(s$expnr1,"_",s$picnr1,"_","centr_measures",".pdf",
sep = ""),
               plot=p, width=10, height=5, dpi=1600)


        p <- ggplot(data = mscr, aes(x = mscrad,fill = coloc_rank))
+
              geom_density(alpha=.2) +
              theme_bw() +
              labs(x="Radius of MSC
[nm]",y="Density",fill='Colocalization rank') +
              ggtitle("Radii of multi-segment complexes based on
their rank") +
              scale_y_continuous(expand = c(0,0)) +
              theme(legend.justification=c(1,0),
```

```
legend.position=c(1,0)) +
              facet_wrap( ~ coloc_rank, ncol=4)

ggsave(file=paste(s$expnr1,"_",s$picnr1,"_","msc_radii",".png", sep
= ""),
               plot=p, width=10, height=5, dpi=1600)

ggsave(file=paste(s$expnr1,"_",s$picnr1,"_","msc_radii",".pdf", sep
= ""),
               plot=p, width=10, height=5, dpi=1600)

        # Look at individual cells
        if (length(unique(msc$cellnr)) > 1) {
          celldf <- NULL
          celldf <- allspots %>% select(cellnr, segment) %>%
            group_by(cellnr,segment) %>%
            summarize(freq = n())
          celldf <- celldf %>% select(cellnr, segment,freq)%>%
            group_by(cellnr) %>%
            mutate(total=sum(freq)) %>%
            ungroup()
          plot_celldf<- celldf%>%
            mutate(cellnr=factor(cellnr)) %>%
            mutate(segment=factor(segment))

          # Clustering
          group_celldf <- spread(celldf, segment, freq)
          group_celldf[is.na(group_celldf)] <- 0
          for (i in 3:10) group_celldf[,i] <- group_celldf[,i]/
group_celldf[2]

          d <- dist(group_celldf[,3:10], method = "euclidean")
          fit <- hclust(d, method="ward.D")
          #plot(fit) # display dendogram
          order <- fit$order
          group_celldf_sorted <- group_celldf[order,]
          group_celldf_sorted <- cbind(group_celldf_sorted,
c(1:length(order)))
          names(group_celldf_sorted)[11] <- "order"
          plot_sorted <- melt(group_celldf_sorted,

id=c("cellnr","total","order"),value.name="segment")
          names(plot_sorted)[4] <- "segment"
          names(plot_sorted)[5] <- "freq"

          # barplot
          fishpalette <- c("#48EBEB", "#99EB48", "#48EB72",
"#EB4848",
                          "#9948EB", "#4872EB", "#EBC048",
"#EB48C0")
          p <- ggplot(subset(plot_sorted,total>cellthresh),aes(x =
order,y = freq)) +
            scale_fill_manual(values=fishpalette,labels=targetnames)
+
```

```
            geom_bar(aes(fill = segment), size =
0,position="fill",stat = "identity")  +
            theme_bw() + coord_flip() +
            xlab('Cell number') +
            ylab('') +
            labs(fill='Segment') +
            scale_y_continuous(expand = c(0,0)) +
            scale_x_discrete(limits=c(1,length(order))) +
            ggtitle("vRNA expression levels vary significantly among
single cells") +
            geom_text(aes(x= order, y= 0.01, label = cellnr,
family="sans", fontface="bold"),
                      size=rel(1.3)) +
            #scale_fill_discrete() +
            theme(axis.text.y=element_blank(),
                  axis.ticks.y=element_blank(),
                  panel.background = element_rect(colour = "black"))

ggsave(file=paste(s$expnr1,"_",s$picnr1,"_","cell_stats_bar3",".png"
, sep = ""),
                 plot=p, width=10, height=8, dpi=1600)

ggsave(file=paste(s$expnr1,"_",s$picnr1,"_","cell_stats_bar3",".pdf"
, sep = ""),
                 plot=p, width=10, height=8, dpi=1600)

          # stripchart
          p <- ggplot(subset(plot_celldf,total>cellthresh),
aes(x=segment, y=freq/total)) +
            geom_boxplot(outlier.shape = NA) +

geom_jitter(aes(color=segment),position=position_jitter(0.4)) +
            scale_color_discrete(labels=targetnames) +
            scale_color_manual(values=fishpalette) +
            labs(color='Segment') +
            xlab('Segment') +
            ylab('Normalized percentage of spots') +
            theme_bw() +
            ggtitle("vRNA expression levels vary significantly among
single cells")
          #stat_summary(fun.y=mean, geom="point", shape=18,
          #             size=3, color="black")

ggsave(file=paste(s$expnr1,"_",s$picnr1,"_","cell_stats_jitter",".pn
g", sep = ""),
                 plot=p, width=10, height=8, dpi=1600)

ggsave(file=paste(s$expnr1,"_",s$picnr1,"_","cell_stats_jitter",".pd
f", sep = ""),
                 plot=p, width=10, height=8, dpi=1600)

          # pie chart
          p <- ggplot(subset(plot_celldf,total>cellthresh),aes(x =
factor(1), y=freq, width=total)) +
```

```
            geom_bar(aes(fill = segment),position = "fill",stat =
"identity")  +
            theme_bw() +
            scale_fill_manual(values=fishpalette,labels=targetnames)
+
            facet_wrap(~cellnr) +
            coord_polar(theta="y") +
            xlab('') +
            ylab('') +
            labs(fill='Segment') +
            ggtitle("vRNA expression levels vary significantly among
single cells") +
            theme(axis.text = element_blank(),
                  axis.ticks = element_blank(),
                  panel.grid  = element_blank(),
                  legend.position="top") +
            geom_text(aes(x= 1, y= 1, label = total, family="sans",
fontface="plain"), size=rel(2), vjust=rel(2.9)) +
            scale_fill_discrete(labels=targetnames)

ggsave(file=paste(s$expnr1,"_",s$picnr1,"_","cell_stats_pie",".png",
sep = ""),
                 plot=p, width=10, height=8, dpi=1600)

ggsave(file=paste(s$expnr1,"_",s$picnr1,"_","cell_stats_pie",".pdf",
sep = ""),
                 plot=p, width=10, height=8, dpi=1600)
        }

        ### Other ggplot2 output
        # MSC density plot
        p <- ggplot() +
          geom_point(aes(x=msc$Pos_X, y=-msc$Pos_Y, alpha =
msc$coloc_rank),size=0.01) +
          theme_bw() +
          labs(x="",y="") +
          scale_fill_discrete(guide=FALSE) +
          guides(size=FALSE) +
          ggtitle("Density of multi-segment complexes shown in z-
projection of image") +
          scale_alpha(guide = 'none') +
          theme(
            axis.text.x = element_blank(),
            axis.text.y = element_blank(),
            axis.ticks = element_blank(),
            panel.grid.major = element_blank(),
            panel.grid.minor = element_blank())

ggsave(file=paste(s$expnr1,"_",s$picnr1,"_","msc_density",".png",
sep = ""),
               plot=p, width=10, height=11, dpi=1600)

ggsave(file=paste(s$expnr1,"_",s$picnr1,"_","msc_density",".pdf",
sep = ""),
```

```
               plot=p, width=10, height=11, dpi=1600)

        # Cell coloured MSC density plot
        p <- ggplot() +
          geom_point(aes(x=msc$Pos_X, y=-msc$Pos_Y, alpha =
msc$coloc_rank, colour = as.factor(msc$cellnr)),size=0.01) +
          theme_bw() +
          labs(x="",y="") +
          scale_fill_discrete(guide=FALSE) +
          guides(size=FALSE, colour=FALSE) +
          ggtitle("Density of multi-segment complexes shown in z-
projection of image") +
          scale_alpha(guide = 'none') +
          theme(
            axis.text.x = element_blank(),
            axis.text.y = element_blank(),
            axis.ticks = element_blank(),
            panel.grid.major = element_blank(),
            panel.grid.minor = element_blank())

ggsave(file=paste(s$expnr1,"_",s$picnr1,"_","msc_density_col",".png"
, sep = ""),
               plot=p, width=10, height=11, dpi=1600)

ggsave(file=paste(s$expnr1,"_",s$picnr1,"_","msc_density_col",".pdf"
, sep = ""),
               plot=p, width=10, height=11, dpi=1600)

        # 8-mers plot for single cells
        plotcells <- c(3,4)
        plotdf_8mers <- msc[msc$cellnr %in% plotcells,]
        plotdf_8mers$coloc_rank <- ifelse(plotdf_8mers$coloc_rank ==
8,1,0.9)
        plotdf_8mers <- plotdf_8mers[!
duplicated(plotdf_8mers$mscnr),]
        p <- ggplot() +
          geom_point(aes(x=plotdf_8mers$Pos_X, y=-
plotdf_8mers$Pos_Y, alpha = plotdf_8mers$coloc_rank,
                         colour =
as.factor(plotdf_8mers$coloc_rank)),size=2) +
          theme_bw() +
          coord_fixed() +
          labs(x="",y="") +
          scale_fill_discrete(guide=FALSE) +
          guides(size=FALSE, colour=FALSE) +
          ggtitle("Location of 8-mer complexes shown in z-projection
of image") +
          scale_alpha(guide = 'none') +
          theme(
            axis.text.x = element_blank(),
            axis.text.y = element_blank(),
            axis.ticks = element_blank(),
            panel.grid.major = element_blank(),
            panel.grid.minor = element_blank())
```

```
ggsave(file=paste(s$expnr1,"_",s$picnr1,"_","msc_8mers",".png", sep
= ""),
               plot=p, width=10, height=11, dpi=400)

ggsave(file=paste(s$expnr1,"_",s$picnr1,"_","msc_8mers",".pdf", sep
= ""),
               plot=p, width=10, height=11, dpi=400)

        # Create scatter plot for all points coloured by segment
        p2 <- ggplot(msc) +
          geom_point(aes(x=Pos_X, y=-Pos_Y, alpha = 0.75, colour =
as.factor(segment)), size=rel(0.01)) +
          theme_bw() +
          labs(x="",y="") +
          guides(size=FALSE)+
          guides(alpha=FALSE)+
          ggtitle("Segment distributions shown in z-projection of
image") +
          labs(colour='Segment') +
          theme(
            axis.text.x = element_blank(),
            axis.text.y = element_blank(),
            axis.ticks = element_blank(),
            panel.grid.major = element_blank(),
            panel.grid.minor = element_blank(),
            legend.position="top") +
          scale_colour_discrete(labels=targetnames)

ggsave(file=paste(s$expnr1,"_",s$picnr1,"_","scatter",".png", sep =
""),
                 plot=p2, width=10, height=11, dpi=1600)

ggsave(file=paste(s$expnr1,"_",s$picnr1,"_","scatter",".pdf", sep =
""),
               plot=p2, width=10, height=11, dpi=1600)

        # TESTING: Scatter test plots for double measurements
        if (s$use_replicates == T) {
          for (seg in 1:segcount) {
          segp <- ggplot(allspots_backup[allspots_backup$segment ==
seg |
                                          allspots_backup$segment
== seg+10,]) +
            geom_point(aes(x=Pos_X, y=-Pos_Y, alpha = 0.75, colour =
as.factor(segment)), size=rel(0.01)) +
            theme_bw() +
            labs(x="",y="") +
            guides(size=FALSE)+
            guides(alpha=FALSE)+
            ggtitle("Double measurements test") +
            labs(colour='Segment') +
            theme(
              axis.text.x = element_blank(),
```

```
                axis.text.y = element_blank(),
                axis.ticks = element_blank(),
                panel.grid.major = element_blank(),
                panel.grid.minor = element_blank(),
                legend.position="top") +
              scale_colour_discrete(labels=c("550","635"))

ggsave(file=paste(s$expnr1,"_",s$picnr1,"_","doublemeasurement_",seg
,".png", sep = ""),
                  plot=segp, width=10, height=11, dpi=160)

ggsave(file=paste(s$expnr1,"_",s$picnr1,"_","doublemeasurement_",seg
,".pdf", sep = ""),
                  plot=segp, width=10, height=11, dpi=160)
          }
        }

        # Optional 3D plotting for data examination
        #scatter3d(x = msc$Pos_X, y = msc$Pos_Y, z = msc$Pos_Z,
surface=F, groups = as.factor(msc$segment),
        #          xlab = "x (nm)", ylab = "y (nm)", zlab = "z
(nm)", axis.scales = F)
        #rgl.viewpoint( theta = 0, phi = -50)
        #par3d("windowRect"= c(0,0,1000,1000))
        #snapshot3d(filename =
paste(s$expnr1,s$picnr1,"3Dplot",".png", sep = "_"))
        #rgl.close()

        # MSC rank distributions plot
        p <- ggplot(msc, aes(x=Pos_X, y=-Pos_Y, colour =
as.factor(coloc_rank))) +
          theme_bw() +
          xlab('X position [nm]') +
          ylab('Y position [nm]') +
          labs(colour='Colocalization rank') +
          scale_x_continuous(expand = c(0,0)) +
          scale_y_continuous(expand = c(0,0)) +
          ggtitle("Distribution of multi-segment complexes based on
their ranks") +
          facet_wrap( ~ coloc_rank, ncol=4) +
          geom_point(size=rel(0.1)) + geom_density2d()

ggsave(file=paste(s$expnr1,"_",s$picnr1,"_","msc_rank_dist",".png",
sep = ""),
               plot=p, width=12, height=8, dpi=1600)

ggsave(file=paste(s$expnr1,"_",s$picnr1,"_","msc_rank_dist",".pdf",
sep = ""),
               plot=p, width=12, height=8, dpi=1600)

        ### END OF GRAPHICAL OUTPUT ###

        ### BEGIN TEXT OUTPUT ###
```

```
        #Number of binary spots containing seg 1 and seg 2
        if (filecount > 1)
          non_normmatrices12 <- matrices_abs[[2]][1,2]

        #Number of binary spots containing seg 1 and seg 3
        if (filecount > 2)
          non_normmatrices13 <- matrices_abs[[2]][1,3]

        #Number of binary spots containing seg 2 and seg 3
        if (filecount > 2)
          non_normmatrices23 <- matrices_abs[[2]][3,2]

        if (virustype == "hanta" && filecount > 2) {
          cat("Seg1&2: ", non_normmatrices12,"\n")
          cat("Seg1&3: ", non_normmatrices13,"\n")
          cat("Seg2&3: ", non_normmatrices23,"\n")
        }

        # Save results as xlsx output file
        split <- str_split(sdir, "/",)
        identifier <- split[[1]][length(split[[1]])-1]

        #xlsxfile <-
paste(s$expnr1,s$picnr1,"Results",identifier,".xlsx", sep = "_")
        xlsxfile <- "Results.xlsx"

        if (file.exists(xlsxfile))
          file.remove(xlsxfile)

        # Main results
        write.xlsx2(amounts,xlsxfile,
                    sheetName = "MainResults", append = TRUE)

        # Write colocalization matrices as absolute values
        write.xlsx2(matrices_abs[[1]],xlsxfile,
                    sheetName = "MatrixAbs_all", append = TRUE)
        write.xlsx2(matrices_abs[[filecount+1]],xlsxfile,
                    sheetName = "MatrixAbs_allbutMonomers", append =
TRUE)
        for (i in 2:filecount) {
          write.xlsx2(matrices_abs[[i]],xlsxfile,
                      sheetName = paste("MatrixAbs",i,sep = "_"),
append = TRUE)
        }

        # Write colocalization matrices as percentages (symmetrical
matrices)
        write.xlsx2(matrices_m[[1]],xlsxfile,
                    sheetName = "Matrix_m_all", append = TRUE)
        write.xlsx2(matrices_m[[filecount+1]],xlsxfile,
                    sheetName = "Matrix_m_allbutMonomers", append =
TRUE)
        for (i in 2:filecount) {
          write.xlsx2(matrices_m[[i]],xlsxfile,
```

```
                      sheetName = paste("Matrix_m",i,sep = "_"),
append = TRUE)
        }

        # Write 2nd color statistics
        if (s$use_replicates == T) {
          write.xlsx2(doublesegstats,xlsxfile,
                      sheetName = "2nd_color_statistics", append =
TRUE)
        }
        # End of xlsx output

        } # end of if-clause: no spots at all

        # Text output HANTA
        if (virustype == "hanta") {
          if (dim(allspots)[1]>0 && filecount > 2) {
            out_line <- paste(strTime, getwd(), df1[1,32], s$xt,
s$yt, s$zt,

nrow(df[[1]]),nrow(df[[2]]),nrow(df[[3]]), #total # seg 1, 2, 3
                              nrow(segment_in_mscs[[1]][[1]]),
nrow(segment_in_mscs[[2]][[1]]), nrow(segment_in_mscs[[3]][[1]]),
#MSC_Sz1: #1, #2, #3
                              non_normmatrices12,
non_normmatrices13, non_normmatrices23, #MSC_Sz2: binary
combinations
                              nrow(segment_in_mscs[[3]][[3]]),sep =
"\t") #MSC_Sz3: # of triplettes
          } else {
            out_line <- paste(strTime, getwd(), srcFileNAme, s$xt,
s$yt, s$zt,
                              0, 0, 0,
                              0, 0, 0,
                              0, 0, 0,
                              0, sep = "\t")
          }

write(out_line,file=paste(mainfolder,"RColoc_Summary.txt",sep=""),ap
pend=TRUE)
        }

        ### END OF TEXT OUTPUT
        strTime <- gsub("-", "", gsub(" ", "",
gsub(":","",Sys.time()))) # Save time of calculation
        cat("Timestamp: ",strTime,"\n") # last line of calculations
    } # of settings-for
} # of filenames-for
```

```
%% data

data = xlsread('SingleCells_nov.xlsx','A2:BR256');
comb = data(:,1);
abun = data(:,2:end);
num_comb=length(comb);

comb_vec=zeros(num_comb,8);
for i=1:length(comb)
    temp = num2str(comb(i));
    for j=1:length(temp)
        temp1=str2num(temp(j));
        comb_vec(i,temp1)=comb_vec(i,temp1)+1;
    end
end

comb_size=zeros(num_comb,1);
for i=1:length(comb)
    comb_size(i)=sum(comb_vec(i,:));
end

num_cells=size(abun,2);

%%
num_cells=size(abun,2);
ind_group=[];
leave_out_k=5;
group_size = ceil(num_cells/leave_out_k);

for i=1:leave_out_k
    ind_group((i-1)*group_size+1:i*group_size) = i;
end
ind_group = ind_group(1:num_cells);

%%

leave_out_k=5;
coef=[];coef_all_r=[];
for loo=1:leave_out_k
    ind_loo = find(ind_group==loo);
    xdata=abun; xdata(:,ind_loo)=[];
    ydata=abun; ydata(:,ind_loo)=[];

    for ii=9:size(xdata,1)
        sizec=nnz(comb_vec(ii,:));
        data_num_all=[];
        for ind_data=1:size(xdata,2)

[param_num,data_num]=calc_num(xdata(:,ind_data),comb_vec,comb_size,i
i);
            if ii<=max(find(comb_size==2))
                param_num=unique(param_num);
                data_num=unique(data_num);
            end
```

```
            par_num=[];
            if ii<=max(find(comb_size==2))
                data_num_all=[data_num_all;data_num];
            else
                len_here=length(param_num)/(sizec);

data_num_all=[data_num_all;data_num(1:len_here:end)];
            end
        end
        coef{loo,ii} =lsqnonneg(data_num_all,ydata(ii,:)');
    end
end

pred_layered=[];
for loo=1:leave_out_k
    ind_loo = find(ind_group==loo);
    for loo1 = 1:length(ind_loo)
        loo2 = ind_loo(loo1);
        for ii=9:size(xdata,1)
            sizec=nnz(comb_vec(ii,:));
            data_num_all_test=[];

[param_num,data_num]=calc_num(abun(:,loo2),comb_vec,comb_size,ii);
            if ii<=max(find(comb_size==2))
                param_num=unique(param_num);
                data_num=unique(data_num);
            end
            par_num=[];
            if ii<=max(find(comb_size==2))
                data_num_all_test=[data_num_all_test;data_num];
            else
                len_here=length(param_num)/(sizec);

data_num_all_test=[data_num_all_test;data_num(1:len_here:end)];
            end
            pred_layered(loo2,ii)=data_num_all_test*coef{loo,ii};
        end
    end
end

%%
range_loo=1:size(abun,2);
layeredk=[];layeredk_r=[];layeredk_a=[];layeredk_all=[];abunk=[];
for kk=2:7
    ind_ii=find(comb_size==kk);
    temp=abun(ind_ii,range_loo)'; temp=temp(:); abunk{kk}=temp;
    temp=pred_layered(range_loo,ind_ii); temp=temp(:); layeredk{kk}
=temp;
end

%%
format short
fonts=20;
ticksize = 15;
```

```
figure
for kk=2:7
    subplot(2,3,kk-1)

    temp1=abunk{kk}(:);
    temp2=layeredk{kk}(:);
    [R,P] = corrcoef(temp1,temp2);
    plot(temp1,temp2,'*')
    s = strcat('K = ',num2str(kk),', ','R = ',sprintf('%0.2f',
round(R(1,2),2)))
    title(s)
    xlim([min([temp1;temp2]),max([temp1;temp2])])
    ylim([min([temp1;temp2]),max([temp1;temp2])])
    set(gca, 'FontSize', ticksize)
end
subplot(2,3,4);xlabel('abundance','fontsize',fonts)
subplot(2,3,5);xlabel('abundance','fontsize',fonts)
subplot(2,3,6);xlabel('abundance','fontsize',fonts)
subplot(2,3,1);ylabel('prediction','fontsize',fonts)
subplot(2,3,4);ylabel('prediction','fontsize',fonts)
```
